# New‐onset autoimmune disease following SARS‐CoV‐2 infection and mRNA vaccination in Norway: A retrospective cohort study

**DOI:** 10.1111/joim.70052

**Published:** 2025-12-02

**Authors:** Håkon Bøås, Paz Lopez‐Doriga Ruiz, Jesper Dahl, Hanne L. Gulseth, German Tapia

**Affiliations:** ^1^ Department of Infection Control and Vaccines Norwegian Institute of Public Health Oslo Norway; ^2^ Department of Chronic Diseases Norwegian Institute of Public Health Oslo Norway; ^3^ Division of Public Health and Prevention Norwegian Institute of Public Health Oslo Norway

**Keywords:** autoimmune disease, COVID‐19, vaccination, SARS‐CoV‐2

## Abstract

**Background:**

Studies suggest an increase in autoimmune diseases following SARS‐CoV‐2 infection and/or COVID‐19‐vaccination. We aimed to describe possible associations in Norway.

**Methods:**

We used information from the emergency preparedness register for COVID‐19, BeredtC19, for all residents aged 18–64 (*N* = 3,450,080). BeredtC19 includes data from mandatory nationwide registers on demographics, SARS‐CoV‐2 tests, deaths, vaccinations, and hospitalizations. Cox regression was used to estimate adjusted hazard ratios (aHRs) for 30‐day and 30–180 day risk windows.

**Findings:**

SARS‐CoV‐2 infection was associated with an increased risk of purpura, thrombocytopenia, and agranulocytosis within 30 days and 30–180 days. In the first 30 days, Bell's palsy and inflammatory arthritis were associated with infection, whereas 30–180 days postinfection showed an association with polyneuropathy. Vaccination was associated with an increased risk of inflammatory bowel disease (IBD) and celiac disease, both in the first 30 days and 30–180 days postvaccination. Additionally, in the first 30 days postvaccination, we found associations with inflammatory arthritis and erythema nodosum, and between 30 and 180 days with arthralgia, agranulocytosis, and acute disseminated encephalomyelitis (ADEM). There was also a reduced risk of dermatopolymyositis 30–180 days postvaccination.

**Interpretation:**

Most autoimmune disorders showed no significant association with SARS‐CoV‐2 infection or COVID‐19 vaccination. Associations between vaccination and IBD and ADEM warrant further investigations, as these observations reflect associations and do not establish causality.

## Graphical abstract

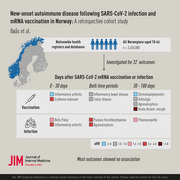

## Introduction

An immune‐mediated response starts when the immune system reacts to a foreign antigen. Abnormal reactions, causing immune‐mediated and autoimmune disease in those genetically predisposed, could potentially be triggered by viral infections [1]. For instance, Epstein–Barr virus has been a suspected agent in multiple sclerosis (MS) [2], and cytomegalovirus has been associated with SLE [3], systemic sclerosis, rheumatoid arthritis (RA), and MS [3].

Vaccines represent one of the most effective public health measures. Nonetheless, concerns have been raised about potential associations between vaccines and immune‐related diseases. Recent examples include Guillain–Barré syndrome [4] and narcolepsy [5] after influenza vaccination. Interestingly, there is a lower risk of idiopathic thrombocytopenic purpura (ITP) after the measles–mumps–rubella (MMR) vaccine than after a natural infection [4], which underlines the importance of investigating both infections and vaccines.

Clinical trials of COVID‐19 mRNA vaccines have reported a slight increase in Bell's palsy after vaccination [6, 7]. As reports of adverse events following immunization (AEFI) arose, the hypothesis that SARS‐CoV‐2 infections or vaccines could cause immune‐mediated diseases gained attention [8]. Although there is a predominance of papers reporting significant associations between SARS‐CoV‐2 infections and autoimmune diseases, there is yet no clear consensus if there is a causal relationship between SARS‐CoV‐2 infection or vaccination and these outcomes. Reported adverse events could either be true AEFIs or might be due to disease debut overlapping with the postimmunization period [9].

Using routinely collected data from mandatory nationwide registers, we aimed to investigate whether the occurrence of immune‐related diseases increased in the first 6 months after a SARS‐CoV‐2 infection or COVID‐19 mRNA vaccination in adults.

## Methods

### Study population

The study sample included all adult Norwegian residents between 18 and 64 years old residing in Norway since January 1, 2017 (*N* = 3,450,080).

### Outcomes and case definition

Based on adverse events reported by health care professionals, mRNA vaccine trials, and Norwegian COVID‐19 surveillance, 32 immune‐mediated outcomes of interest were identified. Table S1 lists all outcomes with ICD‐10 diagnosis codes. A 3‐year wash‐out period (2017–2020) was implemented to exclude prevalent cases (Table S2). Incident outcomes were defined as the first record of the given diagnosis followed by a subsequent record with the same diagnosis within 182 days. Readmissions and hospitalizations <3 days apart were combined.

### Statistical analysis

**Fig. 1:**
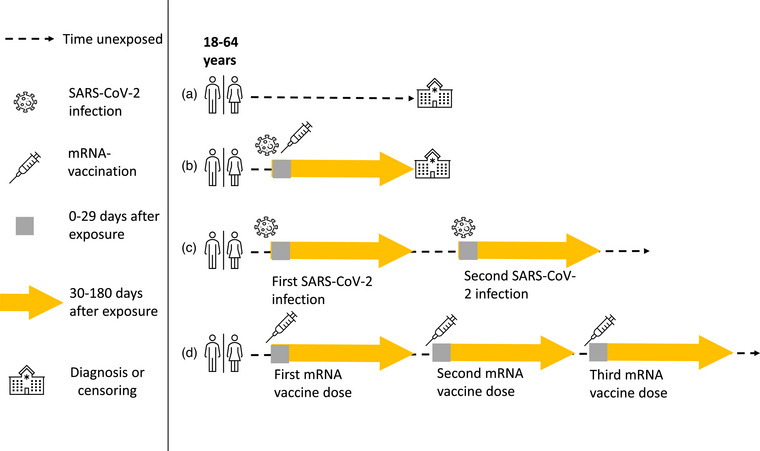
SARS‐CoV‐2 infections and COVID‐19 mRNA vaccination were recorded as exposures. Censoring on date of emigration, death, 65 years of age, non‐mRNA COVID‐19 vaccination, 4th vaccine dose, or occurrence of the outcome. After SARS‐CoV‐2 infection or mRNA vaccination, participants were followed for an immediate risk window of 0–30 days, followed by a 30–180 days window. After 180 days the participants were returned to the unexposed reference group until the next mRNA vaccination or SARS‐CoV‐2 infection occurred. Examples of (a) participant is unexposed and is followed until diagnosis of autoimmune disorder or censoring. (b) Participant diagnosed with autoimmune disorder during risk window after SARS‐CoV‐2 infection or mRNA vaccination. (c and d) Participant was followed to endpoint without development of autoimmune disorder or censoring.

We used registered SARS‐CoV‐2 infections and COVID‐19 mRNA vaccination as time‐varying exposures in a Cox regression model, estimating hazard ratios (HRs) with 95% confidence intervals (95% CI), to assess associations between SARS‐CoV‐2 infection or COVID‐19 mRNA vaccination and selected outcomes. Censoring was done on date of emigration, death, 65 years of age, non‐mRNA COVID‐19 vaccination, 4th vaccine dose, or outcome. We adjusted for age, sex, health region, municipality size, household size, household crowding, low family income, and country of origin. Previous SARS‐CoV‐2 infection was also included and adjusted for in the adjusted vaccination analysis. In our main analysis and in the age‐stratified analyses, all participants were followed for an immediate risk window of 0–30 days, followed by a 30–180‐day window. After 180 days the participants were returned to the unexposed reference group until the next mRNA vaccination or SARS‐CoV‐2 infection occurred (Fig. 1). The first occurrence of an outcome had to be registered by March 1, 2022 for outcomes after infection, and by August 1, 2023 for outcomes after vaccination, to be considered a case. March 1, 2022 was set as the end of follow‐up in the infection analysis to avoid misclassification bias, as test activity later became reduced. Only outcomes with at least 4 events are presented in the main text, with all outcomes presented in Tables S3 and S4. As background incidence rates vary with age [10], and initial vaccine distribution followed age groups by prioritizing elderly and those with underlying diseases [11], we decided to do this study in adults to ensure that individuals had a likelihood of both the exposure and the outcome. We also stratified the analysis on age groups 18–39 (Tables S5 and S6) and 40–64 years old (Tables S7 and S8) to investigate if there were differences between these age‐groups and present a secondary analysis using a 365‐day risk window, for which the first outcome occurrence after vaccination had to be registered by February 1, 2023 (Tables S9 and S10).

### Ethics

Beredt19 use was governed by The Act on Health and Social Preparedness §2–4, granting the Norwegian institute of public health mandate to use registry data for COVID‐19 surveillance and vaccination reporting. The study was approved by the Norwegian Regional Committee for Medical and Health Research Ethics South‐East (REK Sør‐Øst A, ref. 122745).

## Results

**Fig. 2:**
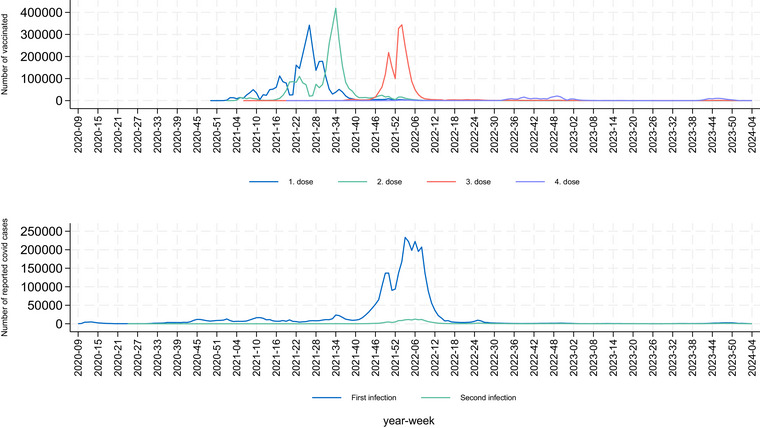
Distribution of vaccine doses (1–4 doses) and reported first and second SARS‐CoV‐2 infection.

Characteristics of the study participants are given in Table 1, 3,450,080 individuals (51% male), with the timely distribution of each vaccine dose and cases of COVID‐19 in the population presented in Fig. 2. The 18–39‐year‐olds were less likely to have received a third dose of a COVID‐19 mRNA vaccine compared to the 40–64‐year‐olds. Most autoimmune disorders were not significantly associated with SARS‐CoV‐2 infections nor COVID‐19 vaccination.

**Table 1:** Demographic characteristics of the study population and the odds ratio (OR) between the age groups and the demographic characteristics.

	Total *N* = 3,450,080 (%)	Age 18–39 *N* = 1,659,446 (48.1%)	Age 40–64 *N* = 1,790,634 (51.9%)	OR (95% CI)^*^
Sex				
Male	1,770,332 (51.3)	854,829 (51.5)	915,503 (51.1)	Ref.
Female	1,679,748 (48.7)	804,617 (48.5)	875,131 (48.9)	1.02 (1.01–1.02)
COVID‐19 infection				
No	2,587,537 (75.0)	1,122,740 (67.7)	1,464,797 (81.8)	
Yes	862,543 (25.0)	536,706 (32.3)	325,837 (18.2)	0.43 (0.46–0.47)
Living conditions				
Cramped	344,407 (10.0)	220,254 (13.3)	124,153 (6.9)	Ref.
Not cramped	3,014,566 (87.4)	1,394,362 (84.0)	1,620,204 (90.5)	2.06 (2.05–2.08)
Unknown	91,107 (2.6)	44,830 (2.7)	46,277 (2.6)	1.83 (1.80–1.86)
Family income				
Normal/Large income	3,002,937 (87.0)	1,361,128 (82.0)	1,641,809 (91.7)	Ref.
Low income	424,473 (12.3)	287,887 (17.4)	136,586 (7.6)	0.39 (0.39–0.40)
Missing	22,670 (0.7)	10,431 (0.6)	12,239 (0.7)	
Origin				
Scandinavia	2,607,560 (75.6)	1,172,876 (70.7)	1,434,684 (80.1)	Ref.
Africa	87,694 (2.5)	57,574 (3.5)	30,120 (1.7)	0.43 (0.42–0.43)
Asia	198,674 (5.8)	121,886 (7.3)	76,788 (4.3)	0.52 (0.51–0.52)
Latin‐America	35,711 (1.0)	21,945 (1.3)	13,766 (0.8)	0.51 (0.51–0.52)
Middle East and North Africa, Sub‐Saharan	63,130 (1.8)	35,581 (2.1)	27,549 (1.5)	0.63 (0.62–0.64)
North America or Oceania	58,757 (1.7)	33,748 (2.0)	25,009 (1.4)	0.61 (0.60–0.62)
Europe	387,434 (11.2)	210,298 (12.7)	177,136 (9.9)	0.69 (0.68–0.69)
Missing	11,120 (0.3)	5538 (0.3)	5582 (0.3)	
Health care region				
Central Regional Health Authority	448,313 (13.0)	216,897 (13.1)	231,416 (12.9)	Ref.
Northern Regional Health Authority	294,527 (8.5)	136,127 (8.2)	158,400 (8.9)	1.09 (1.08–1.10)
South‐Eastern Regional Health Authority	1,945,741 (56.4)	925,791 (55.8)	1,019,950 (57.0)	1.03 (1.03–1.04)
Western Regional Health Authority	699,319 (20.3)	342,295 (20.6)	357,024 (19.9)	0.98 (0.97–0.98)
Missing	62,180 (1.8)	38,336 (2.3)	23,844 (1.3)	
Size of resident county				
<50,000	1,745,488 (50.6)	781,360 (47.1)	964,128 (53.8)	Ref.
≥50,000	1,642,412 (47.6)	839,750 (50.6)	802,662 (44.8)	0.77 (0.77–0.78)
Missing	62,180 (1.8)	38,336 (2.3)	23,884 (1.3)	
Number of vaccinated				
Unvaccinated	334,859 (9.7)	190,643 (11.5)	144,216 (8.1)	Ref.
1 dose	89,833 (2.6)	67,125 (4.1)	22,708 (1.3)	0.45 (0.44–0.45)
2 dose	863,667 (25.0)	617,900 (37.2)	245,767 (13.7)	0.53 (0.52–0.53)
3 dose	2,161,712 (62.7)	783,778 (47.2)	1,377,943 (77.0)	2.32 (2.31–2.34)

Abbreviation: CI, confidence interval.

* *p*‐value < 0.001.

### Neurologic outcomes

**Fig. 3:**
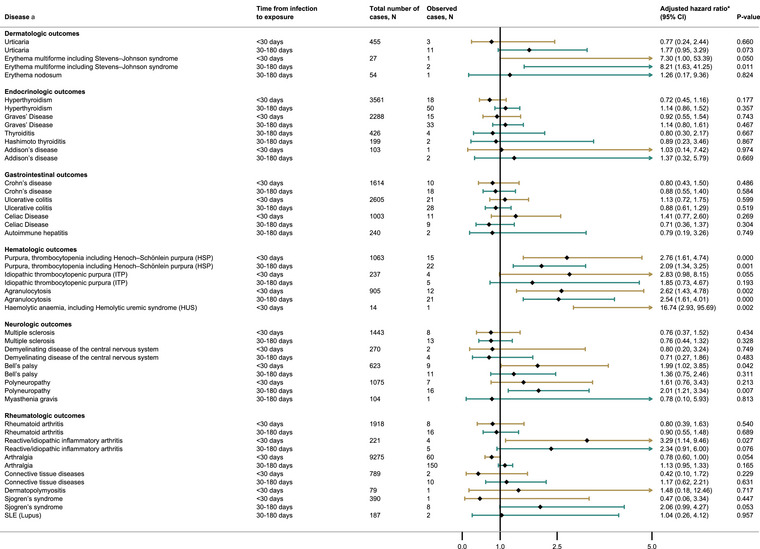
Autoimmune conditions in the population in unexposed and individuals that had one or two SARS‐CoV‐2 infections, using a 0–30 and 30–180 days window of risk. Hazard ratios are adjusted for age, sex, health region, municipality size, household size, household crowding, low family income, and country of origin.

After SARS‐CoV‐2 infection, there were increased risks of Bell's palsy (0–29 days, adjusted hazard ratio [aHR] 2.0 95% CI 1.0–3.9) and polyneuropathy (30–180 days, aHR 2.0 95% CI 1.2–3.4) (Fig. 3). These associations were mainly driven by 18–39‐year‐olds for Bell's palsy (aHR 3.2 95% CI 1.4–7.2) and 40–65‐year‐olds for polyneuropathy (aHR 2.4 95% CI 1.4–4.1) (Tables S5 and S7). Bell's palsy was also associated with infection in the secondary analysis (0–29 days, aHR 2.0 95% CI 1.0–3.8) (Table S9).

**Fig. 4:**
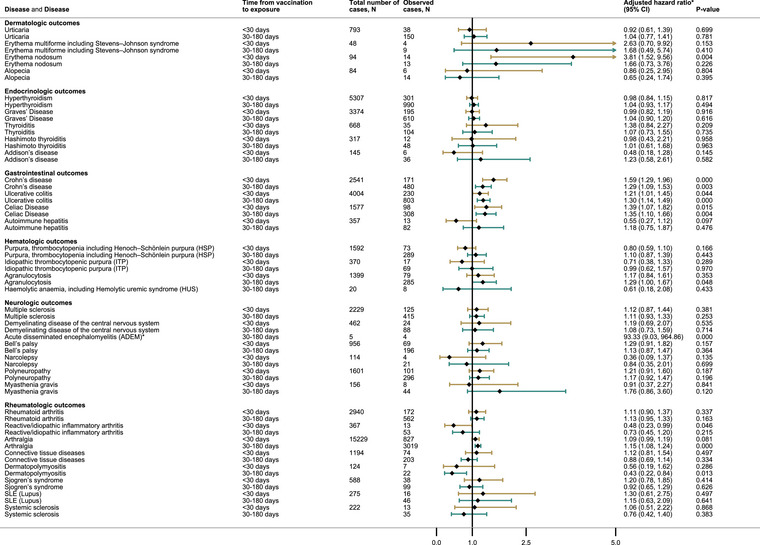
Autoimmune conditions in the population in unexposed and vaccinated individuals using a 0–30 and 30–180 days window of risk. Hazard ratios are adjusted for age, sex, health region, municipality size, household size, household crowding, low family income, country of origin, and previous COVID infection. * For acute disseminated encephalomyelitis (ADEM), the lower boundary of the confidence interval is higher than the upper bounds of the tree.

The only statistically significant association following vaccination was an increase in acute disseminated encephalomyelitis (ADEM) (30–180 days, aHR 93.2, 95% CI 9.0–964.9) (Fig. 4). The number of cases was limited and insufficient for age stratification. In secondary analyses, we found associations after vaccination for Bell's palsy (0–29 days, aHR 1.6, 95% CI 1.1–2.3 and 30–365 days, aHR 1.6, 95% CI 1.2–2.1) and polyneuropathy (0–29 days, aHR 1.5, 95% CI 1.1–2.0 and 30–365 days, aHR 1.7, 95% CI 1.3–2.1, respectively) (Table S10).

### Rheumatologic outcomes

After SARS‐CoV‐2 infection, we observed statistically significant associations with reactive/idiopathic inflammatory arthritis (0–29 days, aHR 3.3 95% CI 1.1–9.5 (Fig. 3), driven by the 40–65‐year‐olds. Among 40–65‐year‐olds, infection was also associated with arthralgia (0–29 days, aHR 0.6 95% CI 0.4–0.9) and Sjogren's syndrome (30–180 days, aHR 2.7 95% CI 1.2–6.2) (Table S7). Among 18–39‐year‐olds, infection was associated with reactive/idiopathic inflammatory arthritis (30–180 days, aHR 3.1 95% CI 1.0–9.3) and arthralgia (30–180 days, aHR 1.3 95% CI 1.0–1.6) (Table S5). In the secondary analysis, reactive/idiopathic inflammatory arthritis was associated with previous SARS‐CoV‐2 infection in both intervals (0–29, aHR 3.3 95% CI 1.2–9.6, and 30–365, aHR 2.2 95% CI 1.0–5.0) (Table S9).

After vaccination, we observed statistically significant associations with reactive/idiopathic inflammatory arthritis (0–29 days, aHR 0.5 95% CI 0.2–1.0), arthralgia (30–180 days, aHR 1.2 95% CI 1.1–1.2), and dermatopolymyositis (30–180 days, aHR 0.4 95% CI 0.2–0.8) (Fig. 4), the last two were mainly driven by the 40–65‐year‐olds (Table S8). In secondary analyses, arthralgia was still associated (0–29 days, aHR 1.1 95% CI 1.0–1.2 and 30–365 days aHR 1.2 95% CI 1.1–1.3), as was RA (30–365 days, aHR 1.3 95% CI 1.1–1.5) (Table S10).

### Endocrinologic outcomes

We did not find any statistically significant associations after SARS‐CoV‐2 infection or vaccination for any endocrinologic outcomes, although only Graves’ disease and hyperthyroidism had sufficient cases for analysis after infection (Figs. 3 and 4).

### Gastrointestinal outcomes

No statistically significant associations with gastrointestinal outcomes were observed after SARS‐CoV‐2 infection (Fig. 3 and Table S3).

After vaccination, we observed associations with Crohn's disease (0–29 days, aHR 1.6 95% CI 1.3–2.0, and 30–180 days, aHR 1.3 95% CI 1.1–1.5), ulcerous colitis (UC) (0–29 days, aHR 1.2 95% CI 1.0–1.4, and 30–180 days, aHR 1.3 95% CI 1.1–1.5), and celiac disease (0–29 days, aHR 1.4 95% CI 1.1–1.8, and 30–180 days, aHR 1.4 95% CI 1.1–1.7) (Fig. 4). In 18–39‐year‐olds, we found associations with Crohn's disease (both intervals) and celiac disease (0–30 days) (Table S6), whereas in 40–65‐year‐olds vaccination was associated with UC (both intervals) and celiac disease (30–180 days) (Table S8). In the secondary analysis, in addition to Crohn's disease, UC, and celiac disease, autoimmune hepatitis was also associated with vaccination (30–365 days, aHR 1.9 95% CI 1.1–3.1) (Table S10).

### Dermatologic outcomes

No statistically significant associations with dermatological outcomes were observed after SARS‐CoV‐2 infection (Fig. 3, Table S3).

Only erythema nodosum had a statistically significant association with vaccination (0–29 days, aHR 3.8 95% CI 1.5–9.6) (Fig. 4), mainly driven by the 40–65‐year‐olds (0–29 days, aHR 5.9 95% CI 1.9–18.6 and 30–180 days, aHR 4.3 95% CI 1.7–11.0 (Table S8).

### Hematologic outcomes

After SARS‐CoV‐2 infections, we found a statistically significant association with purpura, thrombocytopenia including Henoch–Schonlein purpura (HSP) (0–29 days, aHR 2.8 95% CI 1.6–4.7 and 30–180 days, aHR 2.1 95% CI 1.3–3.3), and agranulocytosis (0–29 days, aHR 2.6 95% CI 1.4–4.8 and 30–180 days, aHR 2.5 95% CI 1.6–4.0) (Fig. 3), largely driven by 40–65‐year‐olds (Table S7). The 18–39‐year‐olds were associated with agranulocytosis 30–180 days postinfection (Table S5). The results of the secondary analysis were similar to the primary analysis (Table S9).

We observed an association with agranulocytosis following vaccination (30–180 days, aHR 1.3 95% CI 1.0–1.7) (Fig. 4), driven by the 18–39‐year‐olds (Table S6). In the secondary analysis, agranulocytosis (30–365 days, aHR 1.4 95% CI 1.1–1.8) and purpura, including thrombocytopenia HSP (30–365 days, aHR 1.4 95% CI 1.1–1.8), were associated with vaccination (Table S10).

## Discussion

Most autoimmune disorders were not associated with neither SARS‐CoV‐2 infections nor COVID‐19 mRNA vaccination. Notable exceptions include associations both in the 0–29 days and 30–180 days after infection or vaccination. The risk differed between the 18–39 and 40–64 age groups, as expected, as background incidence rates vary with age [10]. Our study focuses on working‐age adults, and the results are therefore not necessarily transferable to either younger or older populations. The potential underlying mechanisms might differ considerably between different outcomes.

### Strengths and limitations

Norway has universal health care, with free hospital admissions and COVID vaccines. Mandatory registration of COVID‐19 vaccinations and positive SARS‐CoV‐2 tests enabled linkage of nationwide infection and vaccination data to complete population registries, supporting a long wash‐out period (3 years). Despite limited test capacity early in the pandemic [12], testing activity remained high until February 2022 [13], later, proper adjustment for previous COVID‐19 episodes became an increasing challenge, making longer follow‐up times more difficult to attain. Although asymptomatic infections could have remained undetected, high‐quality register data on vaccination and diagnosis codes enabled investigation of several rare outcomes.

Some limitations should be taken into consideration. Rare outcomes could lack statistical power, and the few incident cases of ADEM, hemolytic anemia, and erythema multiforme make a meaningful analysis difficult. The lack of power is more evident for the 0–30 days risk window and the age‐stratified analysis. Results based on a few cases should be interpreted very cautiously. Due to multiple testing, results should be replicated, as we cannot rule out spurious associations. As participants change between exposed and unexposed categories, we did not expect the proportional hazard assumption to be fulfilled. Therefore, the estimated HRs should be interpreted as weighted averages of time‐dependent HRs over the duration of follow‐up [14, 15].

As the percentage of COVID‐19 vaccinated individuals increased, the few unvaccinated individuals might be less representative of the general population. As the unexposed group in this study is comprised both unvaccinated and those vaccinated >180 days earlier (or 365 days earlier in the secondary analysis), we believe this to be less of a problem. As autoimmune diseases can take a long time to develop [16], longer follow up could be needed. Preexisting cases could be misclassified as incident cases. Although the washout period of 3 years should reduce this risk. Capacity problems at the hospitals during the pandemic could postpone consultations [17], likely increasing time between diagnoses. This study was based on hospital data only, and inclusion of primary care diagnoses could potentially have reduced misclassification and increased power for diseases primarily followed up in general practice. Across outcomes, there were only six individuals who did not receive a second diagnosis who died <182 days after the initial diagnosis (not shown). We therefore believe that the slight immortal time from demanding a second diagnosis is justified, as it improves case classification.

We cannot rule out differences between vaccine brands [18] or reverse causality (individuals with unconfirmed autoimmune disease could be more likely to vaccinate), but these factors would mostly affect results the first 0–30 days after vaccination. This study is observational in nature and, as such, is subject to confounding and bias that may not be fully accounted for, despite adjustment for measured covariates. Therefore, the results should be interpreted as associations rather than evidence of direct causal effects.

### Research in context

As the COVID‐19 mRNA vaccines became available, there have been several case reports of incident autoimmune disorders after vaccination and SARS‐CoV‐2 infection [4]. Consequently, a few studies have investigated associations between incident autoimmune disease, SARS‐CoV‐2 infection [19, 20, 21, 22, 23], and COVID‐19 mRNA vaccination [24]. The possibility of developing immune‐mediated diseases following SARS‐CoV‐2 infection or vaccination is not without merit, and some potential mechanisms, for example, molecular mimicry, have been proposed [25]. Differences between the studies often make direct comparisons difficult. As many immune‐mediated diseases develop slowly, the 0–30 days period could unmask prevalent disease, or the challenge of infection or vaccination could cause earlier presentation. Similarly, lockdown and restrictions could have kept individuals from seeking health care. This could lead to an accumulation of cases being diagnosed following a period with many vaccinations or infections. Thus, the etiology of each outcome should be considered when evaluating potential associations.

To allow for a 365‐day risk window in the secondary analysis, the end of follow up before first diagnosis was 6 months earlier than the main analysis. Discrepancies between the primary and secondary analysis should be studied closely to investigate if differences arise due to, for example, incidental finding resulting from loss of power. The longer 365 days risk window could be more susceptible to residual confounding from SARS‐CoV‐2 infections, as test requirements were lifted in the beginning of 2022 [26].

In general, the heterogeneity between published results is large, and some outcomes are only reported in case reports. More studies are needed to put our results in context. See Table S11 for a detailed overview of published literature.

### Neurological disease after SARS‐CoV‐2 infection

Two studies found no increased risk for myasthenia gravis [22, 23], supporting our findings. In contrast, two studies reported an increased occurrence of MS after SARS‐CoV‐2 infections [23, 27], contradicting our result.

Although residual confounding from vaccinations cannot be ruled out, several studies support our findings on Bell's palsy. Patone et al. found an increased risk in the first 2 weeks after infection [28], Li et al. reported higher incidence ratios 90 days postinfection [29], and a study on US veterans found an increase 12 months after infection [30]. These studies suggest an increased risk of Bell's palsy following SARS‐CoV‐2 infection.

### Neurological disease after COVID‐19 mRNA vaccination

We found no comparable studies on MS or other demyelinating diseases of the central nervous system after COVID‐19 mRNA vaccination. In our study, we did not find any increase in MS or other demyelinating diseases following vaccination.

Clinical trials of Comirnaty or Spikevax and The Global COVID Vaccine Safety Project reported a slight increase of Bell's palsy after vaccination [6, 7, 31]. Others found no increased risk [19, 20] or reduced risk following Comirnaty vaccination [29]. We did not find any association with Bell's palsy after COVID‐19 mRNA vaccination in the main analysis, nor when stratifying by age (Tables S6 and S8). In the secondary analysis, there was an increased risk in both the first 30 days and the subsequent 30–365 days after vaccination (Table S10). This result contradicts the main analysis. It is possible that the longer follow‐up time in the secondary analysis provides more power for detecting differences. However, the longer follow‐up time also increases the possibility of incomplete adjustment for asymptomatic SARS‐CoV‐2 infections. Consequently, more studies are needed on the relationship between Bell's palsy and mRNA vaccination.

One study reported more narcolepsy and related disorders after the first Comirnaty dose, but not after the second dose [32]. With only one case 0–30 days after vaccination, the increased risk in 18–39‐year‐olds in our study is likely spurious (Table S6).

The Global COVID Vaccine Safety Project found an increased risk of ADEM in the first 42 days following the first Spikevax dose, but not after subsequent doses [31], whereas others did not find an association [32, 33]. Any ADEM would be expected to occur shortly after exposure. Very few cases, resulting in imprecise estimates, make the validity of our results questionable. Further studies and intercountry collaboration are needed.

### Rheumatologic diseases after SARS‐CoV‐2 infection

Increased risk has been reported for connective tissue diseases [20], Sjogren's syndrome [20, 23], dermatopolymyositis [20], RA [20, 23, 27], SLE [20], and scleroderma [20]. However, other studies found no differences in risk: Sjogren's syndrome [21, 22, 27], dermatopolymyositis [19, 21, 27], RA [21, 22], reactive arthritis [21], SLE [22, 23, 27], and scleroderma [19, 21, 23, 27]. Two studies also reported less SLE among those previously infected [19, 21].

Given the few cases of rheumatic diseases after infection (except for arthralgia), our results should be interpreted cautiously, and there is no clear consensus in the literature on whether SARS‐CoV‐2 infection is associated with rheumatologic diseases.

### Rheumatologic diseases after COVID‐19 mRNA vaccination

Few studies have examined the risk of diseases like connective tissue diseases [24], RA [24, 34], dermatopolymyositis [24], Sjogren's syndrome [24, 32], SLE [24], and scleroderma [24] after vaccination, with most showing no increased risk or even a reduced risk of RA and SLE [32].

One study reported no increased risk of reactive/idiopathic inflammatory arthritis 28 days post vaccination [32], consistent with our results.

### Endocrinologic diseases

The literature on Graves’ disease and Hashimoto's thyroiditis is conflicting, with some studies reporting no change or decreased risk after infection [21, 22, 27, 34], whereas others show an increased risk [23, 27]. Due to the limited number of studies and cases, further research is needed, but a large increase in endocrinologic disease risk after SARS‐CoV‐2 infection or COVID‐19 mRNA vaccination appears unlikely.

### Gastrointestinal diseases

We found no association with inflammatory bowel disease (IBD) and celiac disease after SARS‐CoV‐2 infection. There are several inconsistent studies reporting on IBD risk after SARS‐CoV‐2 infection (increased risk [19, 20, 21, 22, 23], reduced risk [21], or no difference [27, 34]). Results for celiac disease are also inconsistent (no difference in risk [21, 22, 34] and increased risk [20, 23]). No associations for autoimmune hepatitis after SARS‐CoV‐2 infection have been reported [21, 23].

An increased risk of IBD and celiac disease after vaccination was consistently observed in our analysis, but this lacks support in previous literature. Relevant studies have not found any increased risk for IBD [24] or only borderline increased risk of IBD and celiac disease in certain age groups [34].

### Dermatologic diseases

We observed an association with erythema multiforme 30–180 days after SARS‐CoV‐2 infection among the 18–39‐year‐olds. With only three cases in total for both time periods, it is likely that this is spurious association. We found no alopecia cases postinfection; however, most previous literature reports an increase after infection [19, 23].

We were unable to identify other studies investigating most of our dermatologic outcomes after COVID‐19 mRNA vaccination. One study found no increased risk of alopecia among vaccinated [24], supporting our result. Although further studies are needed, we do not see a reason for concern about lasting dermatologic outcomes after COVID‐19 mRNA vaccination.

### Hematologic diseases

The most consistent result among hematologic diseases was an increased risk of purpura, thrombocytopenia including HSP (both periods), and agranulocytosis (both periods) after SARS‐CoV‐2 infection, mainly driven by the 40–65‐year‐olds. Similarly, there was an increased risk of purpura and thrombocytopenia including HSP 30–365 days after vaccination. Given the extended risk window, we cannot rule out residual confounding from unreported SARS‐CoV‐2 infections.

One study found an increased risk of ITP after SARS‐CoV‐2 infection [23], contradicting our result. The Global COVID Vaccine Safety Project found a slight increase in ITP during the first 42 days after vaccination with the first dose of Comirnaty, but not after subsequent doses [31], whereas others did not find an association [32]. From the presented data, there does not seem to be cause for concern about hematologic diseases after COVID‐19 mRNA.

## Conclusions

With the sparsity of published studies on several outcomes, more studies are needed, and rare outcomes should be studied in large international collaborative studies. For most autoimmune disorders, we did not observe any statistically significant association between either of SARS‐CoV‐2 infections or COVID‐19 mRNA vaccination. However, for individual disorders, this study and others have shown some increased disease occurrence after both SARS‐CoV‐2 infection and COVID‐19 mRNA vaccination, although we cannot rule out spurious associations due to multiple testing. Although COVID mRNA vaccines generally should be considered safe, there is a need for more studies, particularly regarding the association between IBD, ADEM, and COVID‐19 mRNA vaccines, as these observations represent associations and do not establish causality.

## Author contributions

All coauthors were involved in the conceptualization of the study, contributed to the interpretation of the results, the revision of the manuscript, and approved the final version for submission. Håkon Bøås, Paz Lopez‐Doriga Ruiz, German Tapia, and Jesper Dahl drafted the study protocol and coordinated the study. Håkon Bøås, German Tapia, and Jesper Dahl contributed directly to the acquisition of data, data cleaning, verification, and preparation and had accessed and verified the final dataset. Håkon Bøås conducted the statistical analysis with support from German Tapia and Jesper Dahl. Håkon Bøås, German Tapia, and Paz Lopez‐Doriga Ruiz conducted the literature search. Håkon Bøås, Paz Lopez‐Doriga Ruiz, and German Tapia drafted the manuscript.

## Conflict of interest statement

PLDR reports participation in research projects funded by pharmaceutical companies, all regulator‐mandated Phase IV studies (PASS) unrelated to the submitted work, with all funds paid to their institution (no personal fees). All other authors report no conflict of interest.

## Funding information

The authors received no funding or grant for this work.

## Supporting information




**Table S1**: Conditions and ICD‐101 codes used to define outcomes of interest.
**Table S2**: Non‐incident and excluded cases for each outcome, because of a 3‐year wash‐out period between 2017 and 2020 to exclude prevalent cases and for those without 2 diagnoses.
**Table S3**: SARS‐CoV‐2 infection and autoimmune conditions (all outcomes) among 18–65‐year‐olds, using a 0–30 and 30–180 days risk window.
**Table S4**: Vaccination and autoimmune conditions (all outcomes) among 18–65‐year‐olds, using a 0–30 and 30–180 days risk window.
**Table S5**: SARS‐CoV‐2 infection and autoimmune conditions (all outcomes) among 18–39‐year‐olds, using a 0–30 and 30–180 days risk window.
**Table S6**: Vaccination and autoimmune conditions (all outcomes) among 18–39‐year‐olds, using a 0–30 and 30–180 days risk window.
**Table S7**: SARS‐CoV‐2 infection and autoimmune conditions (all outcomes) among 40–65‐year‐olds, using a 0–30 and 30–180 days risk window.
**Table S8**: Vaccination and autoimmune conditions (all outcomes) among 40–65‐year‐olds, using a 0–30 and 30–180 days risk window.
**Table S9**: SARS‐CoV‐2 infection and autoimmune conditions (all outcomes) among 18–65‐year‐olds, using a 0–30 and 30–365 days risk window.
**Table S10**: Vaccination and autoimmune conditions (all outcomes) among 18–65‐year‐olds, using a 0–30 and 30–365 days risk window.
**Table S11**: Overview of published literature supporting an association or no association between autoimmune mediated diseases and SARS‐CoV‐2 infection and COVID‐19 mRNA vaccination.

**STROBE Statement** checklist of items that should be included in reports of observational studies,

## Data Availability

The datasets analyzed during the current study come from the national emergency preparedness registry for COVID‐19. The preparedness registry comprised data from a variety of central health registries, national clinical registries, and other national administrative registries. Further information on the preparedness registry is available at https://www.fhi.no/en/id/corona/coronavirus/emergency‐preparedness‐register‐for‐covid‐19/. Access to data from each individual data source is application based through https://helsedata.no/en/, after approval from the Norwegian Committee for Medical and Health Research Ethics and within the framework of the Norwegian data protection legislation.
