# Supplementary material for: New‐onset autoimmune disease following SARS‐CoV‐2 infection and mRNA vaccination in Norway: A retrospective cohort study
Source: J Intern Med. 2025 Dec 2;299(2):271–82. doi: 10.1111/joim.70052 (PMC12789287; doi:10.1111/joim.70052)
Supplement: Supplementary file 1 — Table S1: Conditions and ICD‐101 codes used to define outcomes of interest. Table S2: Non‐incident and excluded cases for each outcome, because of a 3‐year wash‐out period between 2017 and 2020 to exclude prevalent cases and for those without 2 diagnoses. Table S3: SARS‐CoV‐2 infection and autoimmune conditions (all outcomes) among 18–65‐year‐olds, using a 0–30 and 30–180 days risk window. Table S4: Vaccination and autoimmune conditions (all outcomes) among 18–65‐year‐olds, using a 0–30 and 30–180 days risk window. Table S5: SARS‐CoV‐2 infection and autoimmune conditions (all outcomes) among 18–39‐year‐olds, using a 0–30 and 30–180 days risk window. Table S6: Vaccination and autoimmune conditions (all outcomes) among 18–39‐year‐olds, using a 0–30 and 30–180 days risk window. Table S7: SARS‐CoV‐2 infection and autoimmune conditions (all outcomes) among 40–65‐year‐olds, using a 0–30 and 30–180 days risk window. Table S8: Vaccination and autoimmune conditions (all outcomes) among 40–65‐year‐olds, using a 0–30 and 30–180 days risk window. Table S9: SARS‐CoV‐2 infection and autoimmune conditions (all outcomes) among 18–65‐year‐olds, using a 0–30 and 30–365 days risk window. Table S10: Vaccination and autoimmune conditions (all outcomes) among 18–65‐year‐olds, using a 0–30 and 30–365 days risk window. Table S11: Overview of published literature supporting an association or no association between autoimmune mediated diseases and SARS‐CoV‐2 infection and COVID‐19 mRNA vaccination. [file JOIM-299-271-s001.docx]

## Supplemental Material

Table of Content

Supplemental table 1: Conditions and ICD-101 codes used to define outcomes of interest ………. 2

Supplemental table 2: Non-incident and excluded cases for each outcome, because of a 3-year wash-out period between 2017-2020 to exclude prevalent cases and for those without 2 diagnoses ………………………………………………………………………………………………………………………. 3

Supplemental table 3: SARS-CoV-2 infection and autoimmune conditions (all outcomes) among 18–65-year-olds, using a 0-30 and 30-180 days risk window…………………………………………………………………………………………………………………………….. 5

Supplemental table 4: Vaccination and autoimmune conditions (all outcomes) among 18–65-year-olds, using a 0-30 and 30-180 days risk window ……..……………….………………………………. 7

Supplemental table 5: SARS-CoV-2 infection and autoimmune conditions (all outcomes) among 18–39-year-olds, using a 0-30 and 30-180 days risk window ..………………………………. 9

Supplemental table 6: Vaccination and autoimmune conditions (all outcomes) among 18–39-year-olds, using a 0-30 and 30-180 days risk window ……..………………………………………………. 11

Supplemental table 7: SARS-CoV-2 infection and autoimmune conditions (all outcomes) among 40–65-year-olds, using a 0-30 and 30-180 days risk window ………………………………. 13

Supplemental table 8: Vaccination and autoimmune conditions (all outcomes) among 40–65-year-olds, using a 0-30 and 30-180 days risk window …..………………………………………………….15

Supplemental table 9: SARS-CoV-2 infection and autoimmune conditions (all outcomes) among 18–65-year-olds, using a 0-30 and 30-365 days risk window ………………………………. 17

Supplemental table 10: Vaccination and autoimmune conditions (all outcomes) among 18–65-year-olds, using a 0-30 and 30-365 days risk window .……..……………………………………….. 19

Supplemental table 11: Overview of published literature supporting an association or no association between autoimmune mediated diseases and SARS-CoV-2 infection and COVID-19 mRNA vaccination ………………………………………………………………………………………………..…… 21

Supplemental table 1: Conditions and ICD-10 codes used to define outcomes of interest.

| **Condition** | ICD-10^*^ codes |
| --- | --- |
| **Neurologic outcomes** |  |
| Multiple sclerosis | G35 |
| Demyelinating disease of the central nervous system | G36, G37 |
| Acute disseminated encephalomyelitis (ADEM) | G04.0 |
| Bell’s palsy | G51.0 |
| Narcolepsy | G47.4 |
| Polyneuropathy | G61.1, G61.8, G61.9, G62.0, G62.9 |
| Myasthenia gravis | G70.0, G70.9 |
| **Rheumatologic outcomes** |  |
| Rheumatoid arthritis (RA) | M05, M06, M12.3 |
| Reactive/idiopathic inflammatory arthritis | M02.2, M02.3, M02.8, M02.9, M03.6 |
| Arthralgia | M25.5, M25.9 |
| Connective tissue diseases | M32, M33, M34, M35.0, M35.1 |
| Dermatopolymyositis | M33.1, M33.2, M33.9 |
| Sjogren’s syndrome | M35.0 |
| Systemic lupus erythematosus (SLE) | M32 |
| Systemic sclerosis | M34 |
| **Endocrinologic outcomes** |  |
| Hyperthyroidism | E05 |
| Graves’ Disease | E05.0 |
| Thyroiditis | E06.0, E06.1, E06.3, E06.4, E06.9 |
| Hashimoto thyroiditis | E06.3 |
| Addison’s disease | E27.1, E27.2 |
| **Gastrointestinal outcomes** |  |
| Crohn’s disease | K50 |
| Ulcerative colitis (UC) | K51 |
| Celiac disease | K90.0 |
| Autoimmune hepatitis | K75.4 |
| **Dermatologic outcomes** |  |
| Urticaria | L50.1, L50.9 |
| Erythema multiforme including Stevens–Johnson syndrome | L51 |
| Erythema nodosum | L52 |
| Alopecia | L63 |
| **Hematologic outcomes** |  |
| Purpura, thrombocytopenia including Henoch–Schönlein purpura (HSP) | D69.0, D69.2, D69.3, D69.4, D69.5, D69.6, D69.7, D69.8, D69.9 |
| Idiopathic thrombocytopenic purpura (ITP) | D69.3 |
| Agranulocytosis | D70 |
| Haemolytic anaemia, including Hemolytic uremic syndrome (HUS) | D59.0, D59.3 |
| *International Statistical Classification of Diseases and Related Health Problems, Tenth Revision | |

Supplemental table 2. Non-incident and excluded cases for each outcome, because of a 3-year wash-out period between 2017-2020 to exclude prevalent cases and for those without 2 diagnoses.

| **Condition** | **Diagnosis prior to 2020 or before 18 years of age n (%)** | **Initial diagnosis was not repeated within 6 months n (%)** | **Total n (%)** |
| --- | --- | --- | --- |
| **Neurologic outcomes** |  |  |  |
| Multiple sclerosis | 10958 (0.32) | 903 (0.03) | 11861 (0.34) |
| Demyelinating disease of the central nervous system | 1376 (0.04) | 900 (0.03) | 2276 (0.07) |
| Acute disseminated encephalomyelitis (ADEM) | 17 (<0.01) | 19 (<0.01) | 36 (<0.01) |
| Bell’s palsy | 2987 (0.09) | 2889 (0.08) | 5876 (0.17) |
| Narcolepsy | 601 (0.02) | 200 (0.01) | 801 (0.02) |
| Polyneuropathy | 6648 (0.19) | 6287 (0.20) | 13435 (0.39) |
| Myasthenia gravis | 548 (0.02) | 196 (0.01) | 744 (0.02) |
| **Rheumatologic outcomes** |  |  |  |
| Rheumatoid arthritis | 13126 (0.38) | 2469 (0.07) | 15595 (0.45) |
| Reactive/idiopathic inflammatory arthritis | 1609 (0.05) | 879 (0.03) | 2488 (0.07) |
| Arthralgia | 60588 (1.76) | 60056 (1.77) | 120644 (3.50) |
| Connective tissue diseases | 7630 (0.22) | 2716 (0.08) | 10356 (0.30) |
| Dermatopolymyositis | 331 (0.01) | 142 (<0.01) | 473 (0.01) |
| Sjogren’s syndrome | 4169 (0.12) | 2206 (0.06) | 6375 (0.18) |
| SLE (Lupus) | 2280 (0.07) | 305 (0.01) | 2585 (0.07) |
| Systemic sclerosis | 763 (0.02) | 247 (0.01) | 1010 (0.03) |
| **Endocrinologic outcomes** |  |  |  |
| Hyperthyroidism | 10498 (0.3) | 4929 (0.14) | 15427 (0.45) |
| Graves’ Disease | 6574 (0.19) | 3047 (0.09) | 9621 (0.28) |
| Thyroiditis | 2974 (0.09) | 1958 (0.06) | 4932 (0.14) |
| Hashimoto thyroiditis | 1776 (0.05) | 795 (0.02) | 2571 (0.07) |
| Addison’s disease | 902 (0.03) | 240 (0.01) | 1142 (0.03) |
| **Gastrointestinal outcomes** |  |  |  |
| Crohn’s disease | 12552 (0.36) | 2938 (0.09) | 15490 (0.45) |
| Ulcerative colitis | 17798 (0.52) | 5157 (0.15) | 22955 (0.67) |
| Celiac Disease | 7625 (0.22) | 3585 (0.10) | 11210 (0.32) |
| Autoimmune hepatitis | 1341 (0.04) | 321 (0.01) | 1662 (0.05) |
| **Dermatologic outcomes** |  |  |  |
| Urticaria | 2692 (0.08) | 2749 (0.08) | 5441 (0.16) |
| Erythema multiforme including Stevens–Johnson syndrome | 261 (0.01) | 208 (0.01) | 469 (0.01) |
| Erythema nodosum | 383 (0.01) | 297 (0.01) | 680 (0.02) |
| Alopecia | 640 (0.02) | 546 (0.01) | 1186 (0.03) |
| **Hematologic outcomes** |  |  |  |
| Purpura, thrombocytopenia including Henoch–Schönlein purpura (HSP) | 4127 (0.12) | 3708 (0.11) | 7835 (0.23) |
| Idiopathic thrombocytopenic purpura (ITP) | 798 (0.02) | 290 (0.01) | 1088 (0.03) |
| Agranulocytosis | 3755 (0.11) | 3621 (0.11) | 7376 (0.21) |
| Haemolytic anaemia, including Hemolytic uremic syndrome (HUS) | 53 (<0.01) | 45 (<0.01) | 98 (<0.01) |

Supplemental table 3. SARS-CoV-2 infection and autoimmune conditions (all outcomes) among 18–65-year-olds, using a 0-30 and 30-180 days risk window. HR are adjusted for age, sex, health region, municipality size, household size, household crowding, low family income, country of origin.

| **Disease** | **Time from exposure** | **Number of failure** | **Person years at risk** | **Unadjusted** | | **Adjusted** | |
| --- | --- | --- | --- | --- | --- | --- | --- |
|  |  |  |  | **HR (95% CI)** | **p-value** | **HR (95% CI)** | **p-value** |
| **Dermatologic outcomes** | | | | | | | |
| **Urticaria** | **Unexposed** | 441 | 67.94 | Ref. |  | Ref. |  |
|  | **<30 days** | 3 | 0.51 | 0.77 (0.24-2.42) | 0.65 | 0.77 (0.24-2.44) | 0.66 |
|  | **30-180 days ago** | 11 | 0.89 | 1.67 (0.90-3.10) | 0.11 | 1.77 (0.95-3.29) | 0.07 |
| **Erythema multiforme including Stevens–Johnson syndrome** | **Unexposed** | 24 | 68.05 | Ref. |  | Ref. |  |
|  | **<30 days** | 1 | 0.51 | 6.85 (0.98-47.77) | 0.05 | 7.30 (1.00-53.39) | 0.05 |
|  | **30-180 days ago** | 2 | 0.89 | 7.29 (1.56-34.08) | 0.01 | 8.21 (1.63-41.25) | 0.01 |
| **Erythema nodosum** | **Unexposed** | 53 | 68.04 | Ref. |  | Ref. |  |
|  | **<30 days** | 0 | 0.51 | Na | Na | Na | Na |
|  | **30-180 days ago** | 1 | 0.89 | 1.19 (0.17-8.45) | 0.86 | 1.26 (0.17-9.36) | 0.82 |
| **Alopecia** | **Unexposed** | 56 | 68.03 | Ref. |  | Ref. |  |
|  | **<30 days** | 0 | 0.51 | Na | Na | Na | Na |
|  | **30-180 days ago** | 0 | 0.89 | Na | Na | Na | Na |
| **Endocrinologic outcomes** | | | | | | | |
| **Hyperthyroidism** | **Unexposed** | 3493 | 67.70 | Ref. |  | Ref. |  |
|  | **<30 days** | 18 | 0.51 | 0.72 (0.45-1.14) | 0.16 | 0.72 (0.45-1.16) | 0.18 |
|  | **30-180 days ago** | 50 | 0.89 | 1.12 (0.84-1.49) | 0.43 | 1.14 (0.86-1.52) | 0.36 |
| **Graves Disease** | **Unexposed** | 2240 | 67.83 | Ref. |  | Ref. |  |
|  | **<30 days** | 15 | 0.51 | 0.94 (0.56-1.58) | 0.83 | 0.92 (0.55-1.54) | 0.74 |
|  | **30-180 days ago** | 33 | 0.89 | 1.16 (0.82-1.64) | 0.40 | 1.14 (0.80-1.61) | 0.47 |
| **Thyroiditis** | **Unexposed** | 422 | 67.95 | Ref. |  | Ref. |  |
|  | **<30 days** | 0 | 0.51 | Na | Na | Na | Na |
|  | **30-180 days ago** | 4 | 0.89 | 0.88 (0.33-2.39) | 0.81 | 0.80 (0.30-2.17) | 0.67 |
| **Hashimoto thyroiditis** | **Unexposed** | 197 | 68.00 | Ref. |  | Ref. |  |
|  | **<30 days** | 0 | 0.51 | Na | Na | Na | Na |
|  | **30-180 days ago** | 2 | 0.89 | 1.04 (0.27-4.05) | 0.95 | 0.89 (0.23-3.46) | 0.87 |
| **Addison’s disease** | **Unexposed** | 100 | 68.03 | Ref. |  | Ref. |  |
|  | **<30 days** | 1 | 0.51 | 0.88 (0.12-6.28) | 0.90 | 1.03 (0.14-7.42) | 0.97 |
|  | **30-180 days ago** | 2 | 0.89 | 1.14 (0.28-4.67) | 0.86 | 1.37 (0.32-5.79) | 0.67 |
| **Gastrointestinal outcomes** | | | | | | | |
| **Crohn’s disease** | **Unexposed** | 1586 | 67.73 | Ref. |  | Ref. |  |
|  | **<30 days** | 10 | 0.51 | 0.80 (0.43-1.49) | 0.48 | 0.80 (0.43-1.50) | 0.49 |
|  | **30-180 days ago** | 18 | 0.89 | 0.85 (0.53-1.35) | 0.49 | 0.88 (0.55-1.40) | 0.58 |
| **Ulcerative colitis** | **Unexposed** | 2556 | 67.56 | Ref. |  | Ref. |  |
|  | **<30 days** | 21 | 0.51 | 1.12 (0.72-1.74) | 0.61 | 1.13 (0.72-1.75) | 0.60 |
|  | **30-180 days ago** | 28 | 0.89 | 0.85 (0.58-1.23) | 0.39 | 0.88 (0.61-1.29) | 0.52 |
| **Celiac Disease** | **Unexposed** | 983 | 67.84 | Ref. |  | Ref. |  |
|  | **<30 days** | 11 | 0.51 | 1.45 (0.79-2.66) | 0.23 | 1.41 (0.77-2.60) | 0.27 |
|  | **30-180 days ago** | 9 | 0.89 | 0.68 (0.35-1.32) | 0.26 | 0.71 (0.36-1.37) | 0.30 |
| **Autoimmune hepatitis** | **Unexposed** | 238 | 68.02 | Ref. |  | Ref. |  |
|  | **<30 days** | 0 | 0.51 | Na | Na | Na | Na |
|  | **30-180 days ago** | 2 | 0.89 | 0.64 (0.16-2.62) | 0.54 | 0.79 (0.19-3.26) | 0.75 |
| **Hematologic outcomes** | | | | | | | |
| **Purpura, thrombocytopenia including Henoch–Schönlein purpura (HSP)** | **Unexposed** | 1026 | 67.90 | Ref. |  | Ref. |  |
|  | **<30 days** | 15 | 0.51 | 2.28 (1.33-3.91) | 0.00 | 2.76 (1.61-4.74) | 0.00 |
|  | **30-180 days ago** | 22 | 0.89 | 1.78 (1.15-2.76) | 0.01 | 2.09 (1.34-3.25) | 0.00 |
| **Idiopathic thrombocytopenic purpura (ITP)** | **Unexposed** | 228 | 68.03 | Ref. |  | Ref. |  |
|  | **<30 days** | 4 | 0.51 | 2.73 (0.96-7.77) | 0.06 | 2.83 (0.98-8.15) | 0.05 |
|  | **30-180 days ago** | 5 | 0.89 | 1.81 (0.72-4.57) | 0.21 | 1.85 (0.73-4.67) | 0.19 |
| **Agranulocytosis** | **Unexposed** | 872 | 67.91 | Ref. |  | Ref. |  |
|  | **<30 days** | 12 | 0.51 | 1.95 (1.07-3.55) | 0.03 | 2.62 (1.43-4.78) | 0.00 |
|  | **30-180 days ago** | 21 | 0.89 | 1.95 (1.24-3.06) | 0.00 | 2.54 (1.61-4.01) | 0.00 |
| **Haemolytic anaemia, including Hemolytic uremic syndrome (HUS)** | **Unexposed** | 13 | 68.05 | Ref. |  | Ref. |  |
|  | **<30 days** | 1 | 0.51 | 13.09 (2.16-79.53) | 0.01 | 16.74 (2.93-95.69) | 0.00 |
|  | **30-180 days ago** | 0 | 0.89 | Na | Na | Na | Na |
| **Neurologic outcomes** | | | | | | | |
| **Multiple sclerosis** | **Unexposed** | 1422 | 67.80 | Ref. |  | Ref. |  |
|  | **<30 days** | 8 | 0.51 | 0.76 (0.38-1.54) | 0.45 | 0.76 (0.37-1.52) | 0.43 |
|  | **30-180 days ago** | 13 | 0.89 | 0.70 (0.40-1.21) | 0.20 | 0.76 (0.44-1.32) | 0.33 |
| **Demyelinating disease of the central nervous system** | **Unexposed** | 264 | 67.59 | Ref. |  | Ref. |  |
|  | **<30 days** | 2 | 0.51 | 0.84 (0.21-3.40) |  | 0.80 (0.20-3.24) | 0.75 |
|  | **30-180 days ago** | 4 | 1.31 | 0.71 (0.27-1.85) |  | 0.71 (0.27-1.86) | 0.48 |
| **Acute disseminated encephalomyelitis (ADEM)** | **Unexposed** | Na | Na | Ref. |  | Ref. |  |
|  | **<30 days** | Na | Na | Na | NA | Na | NA |
|  | **30-180 days ago** | Na | Na | Na | Na | Na | NA |
| **Bell's palsy** | **Unexposed** | 603 | 67.93 | Ref. |  | Ref. |  |
|  | **<30 days** | 9 | 0.51 | 1.85 (0.95-3.58) | 0.07 | 1.99 (1.02-3.85) | 0.04 |
|  | **30-180 days ago** | 11 | 0.89 | 1.30 (0.72-2.34) | 0.39 | 1.36 (0.75-2.46) | 0.31 |
| **Narcolepsy** | **Unexposed** | 81 | 68.04 | Ref. |  | Ref. |  |
|  | **<30 days** | 0 | 0.51 | Na | Na | Na | Na |
|  | **30-180 days ago** | 0 | 0.89 | Na | Na | Na | Na |
| **Polyneuropathy** | **Unexposed** | 1052 | 67.78 | Ref. |  | Ref. |  |
|  | **<30 days** | 7 | 0.51 | 1.04 (0.49-2.22) | 0.91 | 1.61 (0.76-3.43) | 0.21 |
|  | **30-180 days ago** | 16 | 0.89 | 1.34 (0.81-2.22) | 0.26 | 2.01 (1.21-3.34) | 0.01 |
| **Myasthenia gravis** | **Unexposed** | 103 | 68.04 | Ref. |  | Ref. |  |
|  | **<30 days** | 0 | 0.51 | Na | Na | Na | Na |
|  | **30-180 days ago** | 1 | 0.89 | 0.67 (0.09-5.05) | 0.70 | 0.78 (0.10-5.93) | 0.81 |
| **Rheumatologic outcomes** | | | | | | | |
| **Rheumatoid arthritis** | **Unexposed** | 1894 | 67.72 | Ref. |  | Ref. |  |
|  | **<30 days** | 8 | 0.51 | 0.61 (0.30-1.23) | 0.17 | 0.80 (0.39-1.63) | 0.54 |
|  | **30-180 days ago** | 16 | 0.89 | 0.68 (0.42-1.12) | 0.13 | 0.90 (0.55-1.48) | 0.69 |
| **Reactive/idiopathic inflammatory arthritis** | **Unexposed** | 212 | 68.00 | Ref. |  | Ref. |  |
|  | **<30 days** | 4 | 0.51 | 3.18 (1.13-9.00) | 0.03 | 3.29 (1.14-9.46) | 0.03 |
|  | **30-180 days ago** | 5 | 0.89 | 2.15 (0.84-5.47) | 0.11 | 2.34 (0.91-6.00) | 0.08 |
| **Arthralgia** | **Unexposed** | 9065 | 65.57 | Ref. |  | Ref. |  |
|  | **<30 days** | 60 | 0.49 | 0.79 (0.61-1.02) | 0.07 | 0.78 (0.60-1.00) | 0.05 |
|  | **30-180 days ago** | 150 | 0.86 | 1.16 (0.98-1.37) | 0.08 | 1.13 (0.95-1.33) | 0.17 |
| **Connective tissue diseases** | **Unexposed** | 777 | 67.84 | Ref. |  | Ref. |  |
|  | **<30 days** | 2 | 0.51 | 0.37 (0.09-1.52) | 0.17 | 0.42 (0.10-1.72) | 0.23 |
|  | **30-180 days ago** | 10 | 0.89 | 1.03 (0.55-1.94) | 0.93 | 1.17 (0.62-2.21) | 0.63 |
| **Dermatopolymyositis** | **Unexposed** | 78 | 68.04 | Ref. |  | Ref. |  |
|  | **<30 days** | 1 | 0.51 | 1.31 (0.15-11.22) | 0.81 | 1.48 (0.18-12.46) | 0.72 |
|  | **30-180 days ago** | 0 | 0.89 | Na | Na | Na | Na |
| **Sjogren’s syndrome** | **Unexposed** | 381 | 67.92 | Ref. |  | Ref. |  |
|  | **<30 days** | 1 | 0.51 | 0.37 (0.05-2.66) | 0.32 | 0.47 (0.06-3.34) | 0.45 |
|  | **30-180 days ago** | 8 | 0.89 | 1.64 (0.80-3.38) | 0.18 | 2.06 (0.99-4.27) | 0.05 |
| **SLE (Lupus)** | **Unexposed** | 185 | 68.00 | Ref. |  | Ref. |  |
|  | **<30 days** | 0 | 0.51 | Na | Na | Na | Na |
|  | **30-180 days ago** | 2 | 0.89 | 1.09 (0.28-4.29) | 0.90 | 1.04 (0.26-4.12) | 0.96 |
| **Systemic sclerosis** | **Unexposed** | 147 | 68.03 | Ref. |  | Ref. |  |
|  | **<30 days** | 0 | 0.51 | Na | Na | Na | Na |
|  | **30-180 days ago** | 0 | 0.89 | Na | Na | Na | Na |

Supplemental table 4. Vaccination and autoimmune conditions (all outcomes) among 18–65-year-olds, using a 0-30 and 30-180 days risk window. HR are adjusted for age, sex, health region, municipality size, household size, household crowding, low family income, country of origin, and previous covid infection.

| **Disease** | **Time from exposure** | **Number of failure** | **Person years at risk** | **Unadjusted** | | **Adjusted** | |
| --- | --- | --- | --- | --- | --- | --- | --- |
|  |  |  |  | **HR (95% CI)** | **p-value** | **HR (95% CI)** | **p-value** |
| **Dermatologic outcomes** | | | | | | | |
| **Urticaria** | **Unexposed** | 605 | 84.01 | Ref. |  | Ref. |  |
|  | **<30 days** | 38 | 6.05 | 0.90 (0.60-1.35) | 0.60 | 0.92 (0.61-1.39) | 0.70 |
|  | **30-180 days ago** | 150 | 19.60 | 1.02 (0.76-1.37) | 0.91 | 1.04 (0.77-1.41) | 0.78 |
| **Erythema multiforme including Stevens–Johnson syndrome** | **Unexposed** | 35 | 84.14 | Ref. |  | Ref. |  |
|  | **<30 days** | 4 | 6.06 | 2.33 (0.60-9.07) | 0.22 | 2.63 (0.70-9.92) | 0.15 |
|  | **30-180 days ago** | 9 | 19.63 | 1.51 (0.45-5.11) | 0.51 | 1.68 (0.49-5.74) | 0.41 |
| **Erythema nodosum** | **Unexposed** | 67 | 84.14 | Ref. |  | Ref. |  |
|  | **<30 days** | 14 | 6.06 | 3.77 (1.52-9.37) | 0.00 | 3.81 (1.52-9.56) | 0.00 |
|  | **30-180 days ago** | 13 | 19.63 | 1.53 (0.64-3.67) | 0.34 | 1.66 (0.73-3.76) | 0.23 |
| **Alopecia** | **Unexposed** | 64 | 84.12 | Ref. |  | Ref. |  |
|  | **<30 days** | 6 | 6.06 | 1.00 (0.29-3.46) | 1.00 | 0.86 (0.25-2.95) | 0.80 |
|  | **30-180 days ago** | 14 | 19.63 | 0.77 (0.30-1.97) | 0.58 | 0.65 (0.24-1.74) | 0.39 |
| **Endocrinologic outcomes** | | | | | | | |
| **Hyperthyroidism** | **Unexposed** | 4016 | 83.72 | Ref. |  | Ref. |  |
|  | **<30 days** | 301 | 6.03 | 1.10 (0.94-1.28) | 0.25 | 0.98 (0.84-1.15) | 0.82 |
|  | **30-180 days ago** | 990 | 19.52 | 1.17 (1.04-1.31) | 0.01 | 1.04 (0.93-1.17) | 0.49 |
| **Graves Disease** | **Unexposed** | 2569 | 83.88 | Ref. |  | Ref. |  |
|  | **<30 days** | 195 | 6.04 | 1.07 (0.88-1.29) | 0.51 | 0.99 (0.82-1.19) | 0.92 |
|  | **30-180 days ago** | 610 | 19.56 | 1.13 (0.98-1.31) | 0.10 | 1.04 (0.90-1.20) | 0.62 |
| **Thyroiditis** | **Unexposed** | 529 | 84.03 | Ref. |  | Ref. |  |
|  | **<30 days** | 35 | 6.05 | 1.51 (0.91-2.51) | 0.11 | 1.38 (0.84-2.27) | 0.21 |
|  | **30-180 days ago** | 104 | 19.60 | 1.17 (0.81-1.69) | 0.41 | 1.07 (0.73-1.55) | 0.73 |
| **Hashimoto thyroiditis** | **Unexposed** | 257 | 84.09 | Ref. |  | Ref. |  |
|  | **<30 days** | 12 | 6.06 | 1.15 (0.50-2.60) | 0.75 | 0.98 (0.43-2.21) | 0.96 |
|  | **30-180 days ago** | 48 | 19.62 | 1.17 (0.72-1.90) | 0.52 | 1.01 (0.61-1.68) | 0.96 |
| **Addison’s disease** | **Unexposed** | 103 | 84.13 | Ref. |  | Ref. |  |
|  | **<30 days** | 6 | 6.06 | 0.55 (0.21-1.42) | 0.21 | 0.48 (0.18-1.28) | 0.14 |
|  | **30-180 days ago** | 36 | 19.63 | 1.40 (0.70-2.80) | 0.33 | 1.23 (0.58-2.61) | 0.58 |
| **Gastrointestinal outcomes** | | | | | | | |
| **Crohn’s disease** | **Unexposed** | 1890 | 83.77 | Ref. |  | Ref. |  |
|  | **<30 days** | 171 | 6.03 | 1.54 (1.26-1.89) | 0.00 | 1.59 (1.29-1.96) | 0.00 |
|  | **30-180 days ago** | 480 | 19.53 | 1.24 (1.05-1.45) | 0.01 | 1.29 (1.09-1.53) | 0.00 |
| **Ulcerative colitis** | **Unexposed** | 2971 | 83.57 | Ref. |  | Ref. |  |
|  | **<30 days** | 230 | 6.01 | 1.17 (0.98-1.39) | 0.09 | 1.21 (1.01-1.45) | 0.04 |
|  | **30-180 days ago** | 803 | 19.48 | 1.26 (1.10-1.44) | 0.00 | 1.30 (1.14-1.49) | 0.00 |
| **Celiac Disease** | **Unexposed** | 1171 | 83.88 | Ref. |  | Ref. |  |
|  | **<30 days** | 98 | 6.04 | 1.41 (1.08-1.83) | 0.01 | 1.39 (1.07-1.82) | 0.02 |
|  | **30-180 days ago** | 308 | 19.56 | 1.38 (1.13-1.68) | 0.00 | 1.35 (1.10-1.66) | 0.00 |
| **Autoimmune hepatitis** | **Unexposed** | 262 | 84.11 | Ref. |  | Ref. |  |
|  | **<30 days** | 13 | 6.06 | 0.76 (0.38-1.52) | 0.43 | 0.55 (0.27-1.12) | 0.10 |
|  | **30-180 days ago** | 82 | 19.62 | 1.68 (1.09-2.57) | 0.02 | 1.18 (0.75-1.87) | 0.48 |
| **Hematologic outcomes** | | | | | | | |
| **Purpura, thrombocytopenia including Henoch–Schönlein purpura (HSP)** | **Unexposed** | 1230 | 83.97 | Ref. |  | Ref. |  |
|  | **<30 days** | 73 | 6.05 | 0.85 (0.62-1.16) | 0.30 | 0.80 (0.59-1.10) | 0.17 |
|  | **30-180 days ago** | 289 | 19.59 | 1.16 (0.92-1.46) | 0.22 | 1.10 (0.87-1.39) | 0.44 |
| **Idiopathic thrombocytopenic purpura (ITP)** | **Unexposed** | 284 | 84.12 | Ref. |  | Ref. |  |
|  | **<30 days** | 17 | 6.06 | 0.72 (0.38-1.36) | 0.31 | 0.71 (0.38-1.33) | 0.29 |
|  | **30-180 days ago** | 69 | 19.63 | 1.01 (0.64-1.61) | 0.96 | 0.99 (0.62-1.57) | 0.97 |
| **Agranulocytosis** | **Unexposed** | 1035 | 84.00 | Ref. |  | Ref. |  |
|  | **<30 days** | 79 | 6.05 | 1.22 (0.88-1.68) | 0.23 | 1.17 (0.84-1.61) | 0.35 |
|  | **30-180 days ago** | 285 | 19.59 | 1.38 (1.08-1.77) | 0.01 | 1.29 (1.00-1.67) | 0.05 |
| **Haemolytic anaemia, including Hemolytic uremic syndrome (HUS)** | **Unexposed** | 12 | 84.15 | Ref. |  | Ref. |  |
|  | **<30 days** | 0 | 6.06 | Na | Na | Na | Na |
|  | **30-180 days ago** | 8 | 19.63 | 0.69 (0.21-2.29) | 0.55 | 0.61 (0.18-2.08) | 0.43 |
| **Neurologic outcomes** | | | | | | | |
| **Multiple sclerosis** | **Unexposed** | 1689 | 83.87 | Ref. |  | Ref. |  |
|  | **<30 days** | 125 | 6.03 | 1.15 (0.90-1.47) | 0.25 | 1.12 (0.87-1.44) | 0.38 |
|  | **30-180 days ago** | 415 | 19.55 | 1.15 (0.97-1.37) | 0.12 | 1.11 (0.93-1.33) | 0.25 |
| **Demyelinating disease of the central nervous system** | **Unexposed** | 350 | 84.09 | Ref. |  | Ref. |  |
|  | **<30 days** | 24 | 6.06 | 1.24 (0.72-2.15) |  | 1.19 (0.69-2.07) | 0.53 |
|  | **30-180 days ago** | 88 | 19.62 | 1.12 (0.76-1.63) |  | 1.08 (0.73-1.59) | 0.71 |
| **Acute disseminated encephalomyelitis (ADEM)** | **Unexposed** | 1 | 84.15 | Ref. |  | Ref. |  |
|  | **<30 days** | 0 | 6.06 | Na | Na | Na | Na |
|  | **30-180 days ago** | 4 | 19.63 | 89.61 (9.57-838.86) | 0.25 | 93.33 (9.03-964.86) | 0.00 |
| **Bell's palsy** | **Unexposed** | 691 | 84.00 | Ref. |  | Ref. |  |
|  | **<30 days** | 69 | 6.05 | 1.35 (0.95-1.91) | 0.09 | 1.29 (0.91-1.82) | 0.16 |
|  | **30-180 days ago** | 196 | 19.60 | 1.19 (0.92-1.54) | 0.18 | 1.13 (0.87-1.47) | 0.36 |
| **Narcolepsy** | **Unexposed** | 89 | 84.13 | Ref. |  | Ref. |  |
|  | **<30 days** | 4 | 6.06 | 0.38 (0.11-1.31) | 0.12 | 0.36 (0.09-1.37) | 0.13 |
|  | **30-180 days ago** | 21 | 19.63 | 0.84 (0.38-1.82) | 0.65 | 0.84 (0.35-2.01) | 0.70 |
| **Polyneuropathy** | **Unexposed** | 1204 | 83.85 | Ref. |  | Ref. |  |
|  | **<30 days** | 101 | 6.03 | 1.58 (1.19-2.08) | 0.00 | 1.21 (0.91-1.60) | 0.19 |
|  | **30-180 days ago** | 296 | 19.55 | 1.53 (1.23-1.92) | 0.00 | 1.17 (0.92-1.47) | 0.20 |
| **Myasthenia gravis** | **Unexposed** | 104 | 84.13 | Ref. |  | Ref. |  |
|  | **<30 days** | 8 | 6.06 | 1.04 (0.42-2.58) | 0.94 | 0.91 (0.37-2.27) | 0.84 |
|  | **30-180 days ago** | 44 | 19.63 | 2.00 (0.98-4.12) | 0.06 | 1.76 (0.86-3.60) | 0.12 |
| **Rheumatologic outcomes** | | | | | | | |
| **Rheumatoid arthritis** | **Unexposed** | 2206 | 83.80 | Ref. |  | Ref. |  |
|  | **<30 days** | 172 | 6.03 | 1.43 (1.16-1.76) | 0.00 | 1.11 (0.90-1.37) | 0.34 |
|  | **30-180 days ago** | 562 | 19.53 | 1.46 (1.24-1.71) | 0.00 | 1.13 (0.95-1.33) | 0.16 |
| **Reactive/idiopathic inflammatory arthritis** | **Unexposed** | 301 | 84.09 | Ref. |  | Ref. |  |
|  | **<30 days** | 13 | 6.06 | 0.48 (0.24-0.97) | 0.04 | 0.48 (0.23-0.99) | 0.05 |
|  | **30-180 days ago** | 53 | 19.62 | 0.72 (0.46-1.15) | 0.17 | 0.73 (0.45-1.20) | 0.22 |
| **Arthralgia** | **Unexposed** | 11383 | 81.10 | Ref. |  | Ref. |  |
|  | **<30 days** | 827 | 5.83 | 1.20 (1.09-1.31) | 0.00 | 1.09 (0.99-1.19) | 0.08 |
|  | **30-180 days ago** | 3019 | 18.87 | 1.26 (1.18-1.35) | 0.00 | 1.15 (1.08-1.24) | 0.00 |
| **Connective tissue diseases** | **Unexposed** | 917 | 83.91 | Ref. |  | Ref. |  |
|  | **<30 days** | 74 | 6.04 | 1.34 (0.96-1.85) | 0.08 | 1.12 (0.81-1.54) | 0.50 |
|  | **30-180 days ago** | 203 | 19.57 | 1.07 (0.83-1.37) | 0.60 | 0.88 (0.69-1.14) | 0.33 |
| **Dermatopolymyositis** | **Unexposed** | 95 | 84.14 | Ref. |  | Ref. |  |
|  | **<30 days** | 7 | 6.06 | 0.71 (0.25-1.98) | 0.51 | 0.56 (0.19-1.62) | 0.29 |
|  | **30-180 days ago** | 22 | 19.63 | 0.60 (0.31-1.16) | 0.13 | 0.43 (0.22-0.84) | 0.01 |
| **Sjogren’s syndrome** | **Unexposed** | 451 | 84.00 | Ref. |  | Ref. |  |
|  | **<30 days** | 38 | 6.05 | 1.44 (0.92-2.25) | 0.11 | 1.20 (0.78-1.85) | 0.41 |
|  | **30-180 days ago** | 99 | 19.59 | 1.10 (0.78-1.55) | 0.58 | 0.92 (0.65-1.29) | 0.63 |
| **SLE (Lupus)** | **Unexposed** | 213 | 84.09 | Ref. |  | Ref. |  |
|  | **<30 days** | 16 | 6.06 | 1.35 (0.64-2.86) | 0.43 | 1.30 (0.61-2.75) | 0.50 |
|  | **30-180 days ago** | 46 | 19.62 | 1.22 (0.68-2.18) | 0.51 | 1.15 (0.63-2.09) | 0.64 |
| **Systemic sclerosis** | **Unexposed** | 174 | 84.13 | Ref. |  | Ref. |  |
|  | **<30 days** | 13 | 6.06 | 1.48 (0.70-3.15) | 0.31 | 1.06 (0.51-2.22) | 0.87 |
|  | **30-180 days ago** | 35 | 19.63 | 1.18 (0.64-2.17) | 0.59 | 0.76 (0.42-1.40) | 0.38 |

Supplemental table 5. SARS-CoV-2 infection and autoimmune conditions (all outcomes) among 18–39-year-olds, using a 0-30 and 30-180 days risk window. HR are adjusted for age, sex, health region, municipality size, household size, household crowding, low family income, country of origin.

| **Disease** | **Time from exposure** | **Number of failure** | **Person years at risk** | **Unadjusted** | | **Adjusted** | |
| --- | --- | --- | --- | --- | --- | --- | --- |
|  |  |  |  | **HR (95% CI)** | **p-value** | **HR (95% CI)** | **p-value** |
| **Dermatologic outcomes** | | | | | | | |
| **Urticaria** | **Unexposed** | 227 | 31.34 | Ref. |  | Ref. |  |
|  | **<30 days** | 1 | 0.31 | 0.34 (0.05-2.42) | 0.28 | 0.36 (0.05-2.53) | 0.30 |
|  | **30-180 days ago** | 7 | 0.52 | 1.51 (0.68-3.35) | 0.31 | 1.64 (0.74-3.64) | 0.22 |
| **Erythema multiforme including Stevens–Johnson syndrome** | **Unexposed** | 11 | 31.39 | Ref. |  | Ref. |  |
|  | **<30 days** | 1 | 0.31 | 10.99 (1.96-61.77) | 0.01 | 13.23 (2.19-79.99) | 0.00 |
|  | **30-180 days ago** | 2 | 0.52 | 12.22 (2.74-54.51) | 0.00 | 15.25 (2.97-78.43) | 0.00 |
| **Erythema nodosum** | **Unexposed** | 29 | 31.39 | Ref. |  | Ref. |  |
|  | **<30 days** | 0 | 0.31 | Na | Na | Na | Na |
|  | **30-180 days ago** | 1 | 0.52 | 1.81 (0.24-13.69) | 0.56 | 2.18 (0.27-17.85) | 0.47 |
| **Alopecia** | **Unexposed** | 32 | 31.38 | Ref. |  | Ref. |  |
|  | **<30 days** | 0 | 0.31 | Na | Na | Na | Na |
|  | **30-180 days ago** | 0 | 0.52 | Na | Na | Na | Na |
| **Endocrinologic outcomes** | | | | | | | |
| **Hyperthyroidism** | **Unexposed** | 1327 | 31.28 | Ref. |  | Ref. |  |
|  | **<30 days** | 9 | 0.31 | 0.75 (0.38-1.46) | 0.40 | 0.71 (0.36-1.39) | 0.32 |
|  | **30-180 days ago** | 26 | 0.52 | 1.25 (0.85-1.86) | 0.26 | 1.23 (0.83-1.83) | 0.30 |
| **Graves Disease** | **Unexposed** | 920 | 31.32 | Ref. |  | Ref. |  |
|  | **<30 days** | 7 | 0.31 | 0.82 (0.38-1.77) | 0.61 | 0.77 (0.36-1.66) | 0.50 |
|  | **30-180 days ago** | 19 | 0.52 | 1.28 (0.81-2.02) | 0.29 | 1.24 (0.79-1.96) | 0.35 |
| **Thyroiditis** | **Unexposed** | 169 | 31.36 | Ref. |  | Ref. |  |
|  | **<30 days** | 0 | 0.31 | Na | Na | Na | Na |
|  | **30-180 days ago** | 4 | 0.52 | 1.91 (0.69-5.29) | 0.21 | 1.68 (0.60-4.72) | 0.33 |
| **Hashimoto thyroiditis** | **Unexposed** | 98 | 31.37 | Ref. |  | Ref. |  |
|  | **<30 days** | 0 | 0.31 | Na | Na | Na | Na |
|  | **30-180 days ago** | 2 | 0.52 | 1.66 (0.44-6.32) | 0.46 | 1.43 (0.37-5.49) | 0.60 |
| **Addison’s disease** | **Unexposed** | 40 | 31.39 | Ref. |  | Ref. |  |
|  | **<30 days** | 1 | 0.31 | 1.20 (0.16-9.09) | 0.86 | 1.25 (0.16-9.64) | 0.83 |
|  | **30-180 days ago** | 1 | 0.52 | 0.87 (0.10-7.40) | 0.90 | 0.94 (0.11-8.47) | 0.96 |
| **Gastrointestinal outcomes** | | | | | | | |
| **Crohn’s disease** | **Unexposed** | 833 | 31.26 | Ref. |  | Ref. |  |
|  | **<30 days** | 8 | 0.31 | 0.90 (0.44-1.82) | 0.77 | 0.92 (0.45-1.87) | 0.81 |
|  | **30-180 days ago** | 14 | 0.51 | 0.96 (0.56-1.63) | 0.87 | 0.99 (0.58-1.69) | 0.97 |
| **Ulcerative colitis** | **Unexposed** | 1417 | 31.20 | Ref. |  | Ref. |  |
|  | **<30 days** | 15 | 0.30 | 1.03 (0.61-1.74) | 0.91 | 1.10 (0.65-1.87) | 0.72 |
|  | **30-180 days ago** | 21 | 0.51 | 0.86 (0.56-1.32) | 0.49 | 0.95 (0.61-1.46) | 0.81 |
| **Celiac Disease** | **Unexposed** | 569 | 31.28 | Ref. |  | Ref. |  |
|  | **<30 days** | 8 | 0.31 | 1.27 (0.62-2.60) | 0.52 | 1.32 (0.64-2.71) | 0.45 |
|  | **30-180 days ago** | 8 | 0.51 | 0.76 (0.38-1.55) | 0.45 | 0.84 (0.41-1.72) | 0.64 |
| **Autoimmune hepatitis** | **Unexposed** | 69 | 31.39 | Ref. |  | Ref. |  |
|  | **<30 days** | 0 | 0.31 | Na | Na | Na | Na |
|  | **30-180 days ago** | 1 | 0.52 | 0.73 (0.10-5.36) | 0.76 | 0.76 (0.10-5.59) | 0.79 |
| **Hematologic outcomes** | | | | | | | |
| **Purpura, thrombocytopenia including Henoch–Schönlein purpura (HSP)** | **Unexposed** | 339 | 31.34 | Ref. |  | Ref. |  |
|  | **<30 days** | 5 | 0.31 | 1.76 (0.68-4.59) | 0.25 | 1.73 (0.66-4.54) | 0.26 |
|  | **30-180 days ago** | 8 | 0.52 | 1.60 (0.77-3.31) | 0.20 | 1.60 (0.76-3.36) | 0.21 |
| **Idiopathic thrombocytopenic purpura (ITP)** | **Unexposed** | 100 | 31.39 | Ref. |  | Ref. |  |
|  | **<30 days** | 2 | 0.31 | 2.29 (0.49-10.80) | 0.29 | 2.12 (0.44-10.18) | 0.35 |
|  | **30-180 days ago** | 3 | 0.52 | 1.98 (0.59-6.71) | 0.27 | 1.84 (0.54-6.32) | 0.33 |
| **Agranulocytosis** | **Unexposed** | 230 | 31.36 | Ref. |  | Ref. |  |
|  | **<30 days** | 3 | 0.31 | 1.55 (0.46-5.25) | 0.48 | 1.60 (0.47-5.43) | 0.45 |
|  | **30-180 days ago** | 8 | 0.52 | 2.36 (1.14-4.89) | 0.02 | 2.38 (1.13-5.01) | 0.02 |
| **Haemolytic anaemia, including Hemolytic uremic syndrome (HUS)** | **Unexposed** | 5 | 31.40 | Ref. |  | Ref. |  |
|  | **<30 days** | 0 | 0.31 | Na | Na | Na | Na |
|  | **30-180 days ago** | 0 | 0.52 | Na | Na | Na | Na |
| **Neurologic outcomes** | | | | | | | |
| **Multiple sclerosis** | **Unexposed** | 742 | 31.33 | Ref. |  | Ref. |  |
|  | **<30 days** | 8 | 0.31 | 1.09 (0.53-2.22) | 0.82 | 1.15 (0.56-2.36) | 0.70 |
|  | **30-180 days ago** | 10 | 0.52 | 0.80 (0.43-1.50) | 0.49 | 0.92 (0.49-1.74) | 0.80 |
| **Demyelinating disease of the central nervous system** | **Unexposed** | 114 | 31.38 | Ref. |  | Ref. |  |
|  | **<30 days** | 1 | 0.31 | 0.71 (0.10-5.11) |  | 0.71 (0.10-5.12) | 0.73 |
|  | **30-180 days ago** | 2 | 0.52 | 0.88 (0.23-3.29) |  | 0.88 (0.23-3.32) | 0.85 |
| **Acute disseminated encephalomyelitis (ADEM)** | **Unexposed** | NA | NA | Ref. |  | Ref. |  |
|  | **<30 days** | NA | NA | Na | NA | Na | NA |
|  | **30-180 days ago** | NA | NA | Na | Na | Na | NA |
| **Bell's palsy** | **Unexposed** | 212 | 31.35 | Ref. |  | Ref. |  |
|  | **<30 days** | 6 | 0.31 | 3.25 (1.44-7.34) | 0.00 | 3.17 (1.40-7.22) | 0.01 |
|  | **30-180 days ago** | 5 | 0.52 | 1.48 (0.64-3.43) | 0.35 | 1.46 (0.63-3.41) | 0.38 |
| **Narcolepsy** | **Unexposed** | 58 | 31.39 | Ref. |  | Ref. |  |
|  | **<30 days** | 0 | 0.31 | Na | Na | Na | Na |
|  | **30-180 days ago** | 0 | 0.52 | Na | Na | Na | Na |
| **Polyneuropathy** | **Unexposed** | 153 | 31.36 | Ref. |  | Ref. |  |
|  | **<30 days** | 1 | 0.31 | 0.93 (0.12-7.21) | 0.94 | 1.01 (0.13-7.94) | 0.99 |
|  | **30-180 days ago** | 1 | 0.52 | 0.51 (0.07-3.67) | 0.50 | 0.59 (0.08-4.19) | 0.60 |
| **Myasthenia gravis** | **Unexposed** | 27 | 31.39 | Ref. |  | Ref. |  |
|  | **<30 days** | 0 | 0.31 | Na | Na | Na | Na |
|  | **30-180 days ago** | 0 | 0.52 | Na | Na | Na | Na |
| **Rheumatologic outcomes** | | | | | | | |
| **Rheumatoid arthritis** | **Unexposed** | 460 | 31.34 | Ref. |  | Ref. |  |
|  | **<30 days** | 3 | 0.31 | 0.83 (0.25-2.69) | 0.75 | 0.88 (0.27-2.86) | 0.83 |
|  | **30-180 days ago** | 7 | 0.52 | 1.03 (0.48-2.23) | 0.94 | 1.20 (0.56-2.61) | 0.64 |
| **Reactive/idiopathic inflammatory arthritis** | **Unexposed** | 113 | 31.37 | Ref. |  | Ref. |  |
|  | **<30 days** | 0 | 0.31 | Na | Na | Na | Na |
|  | **30-180 days ago** | 4 | 0.52 | 2.82 (0.95-8.35) | 0.06 | 3.13 (1.05-9.33) | 0.04 |
| **Arthralgia** | **Unexposed** | 3543 | 30.47 | Ref. |  | Ref. |  |
|  | **<30 days** | 37 | 0.30 | 0.97 (0.69-1.35) | 0.84 | 0.92 (0.66-1.30) | 0.65 |
|  | **30-180 days ago** | 82 | 0.50 | 1.30 (1.04-1.62) | 0.02 | 1.28 (1.02-1.61) | 0.03 |
| **Connective tissue diseases** | **Unexposed** | 258 | 31.35 | Ref. |  | Ref. |  |
|  | **<30 days** | 0 | 0.31 | Na | Na | Na | Na |
|  | **30-180 days ago** | 2 | 0.52 | 0.43 (0.10-1.82) | 0.25 | 0.42 (0.10-1.80) | 0.24 |
| **Dermatopolymyositis** | **Unexposed** | 27 | 31.40 | Ref. |  | Ref. |  |
|  | **<30 days** | 0 | 0.31 | Na | Na | Na | Na |
|  | **30-180 days ago** | 0 | 0.52 | Na | Na | Na | Na |
| **Sjogren’s syndrome** | **Unexposed** | 96 | 31.37 | Ref. |  | Ref. |  |
|  | **<30 days** | 0 | 0.31 | Na | Na | Na | Na |
|  | **30-180 days ago** | 2 | 0.52 | 0.96 (0.20-4.54) | 0.96 | 0.96 (0.20-4.69) | 0.96 |
| **SLE (Lupus)** | **Unexposed** | 92 | 31.38 | Ref. |  | Ref. |  |
|  | **<30 days** | 0 | 0.31 | Na | Na | Na | Na |
|  | **30-180 days ago** | 0 | 0.52 | Na | Na | Na | Na |
| **Systemic sclerosis** | **Unexposed** | 28 | 31.39 | Ref. |  | Ref. |  |
|  | **<30 days** | 0 | 0.31 | Na | Na | Na | Na |
|  | **30-180 days ago** | 0 | 0.52 | Na | Na | Na | Na |

Supplemental table 6. Vaccination and autoimmune conditions (all outcomes) among 18–39-year-olds, using a 0-30 and 30-180 days risk window. HR are adjusted for age, sex, health region, municipality size, household size, household crowding, low family income, country of origin, and previous covid infection.

| **Disease** | **Time from exposure** | **Number of failure** | **Person years at risk** | **Unadjusted** | | **Adjusted** | |
| --- | --- | --- | --- | --- | --- | --- | --- |
|  |  |  |  | **HR (95% CI)** | **p-value** | **HR (95% CI)** | **p-value** |
| **Dermatologic outcomes** | | | | | | | |
| **Urticaria** | **Unexposed** | 328 | 40.59 | Ref. |  | Ref. |  |
|  | **<30 days** | 21 | 2.59 | 1.07 (0.63-1.81) | 0.81 | 1.00 (0.59-1.70) | 0.99 |
|  | **30-180 days ago** | 74 | 8.23 | 1.08 (0.71-1.64) | 0.71 | 1.01 (0.67-1.53) | 0.96 |
| **Erythema multiforme including Stevens–Johnson syndrome** | **Unexposed** | 19 | 40.66 | Ref. |  | Ref. |  |
|  | **<30 days** | 1 | 2.59 | 1.73 (0.16-18.43) | 0.65 | 2.20 (0.23-21.15) | 0.49 |
|  | **30-180 days ago** | 5 | 8.25 | 2.45 (0.79-7.61) | 0.12 | 2.99 (0.88-10.15) | 0.08 |
| **Erythema nodosum** | **Unexposed** | 50 | 40.65 | Ref. |  | Ref. |  |
|  | **<30 days** | 7 | 2.59 | 3.18 (0.83-12.24) | 0.09 | 2.91 (0.73-11.55) | 0.13 |
|  | **30-180 days ago** | 4 | 8.25 | 0.72 (0.20-2.56) | 0.61 | 0.62 (0.17-2.24) | 0.47 |
| **Alopecia** | **Unexposed** | 35 | 40.65 | Ref. |  | Ref. |  |
|  | **<30 days** | 4 | 2.59 | 1.71 (0.28-10.45) | 0.56 | 1.61 (0.26-9.79) | 0.61 |
|  | **30-180 days ago** | 5 | 8.25 | 0.78 (0.17-3.62) | 0.75 | 0.69 (0.14-3.42) | 0.65 |
| **Endocrinologic outcomes** | | | | | | | |
| **Hyperthyroidism** | **Unexposed** | 1599 | 40.50 | Ref. |  | Ref. |  |
|  | **<30 days** | 90 | 2.58 | 0.81 (0.62-1.07) | 0.14 | 0.77 (0.59-1.01) | 0.06 |
|  | **30-180 days ago** | 329 | 8.21 | 1.11 (0.92-1.34) | 0.27 | 1.05 (0.87-1.26) | 0.63 |
| **Graves Disease** | **Unexposed** | 1094 | 40.56 | Ref. |  | Ref. |  |
|  | **<30 days** | 68 | 2.59 | 0.92 (0.66-1.28) | 0.63 | 0.88 (0.64-1.21) | 0.44 |
|  | **30-180 days ago** | 233 | 8.23 | 1.22 (0.97-1.54) | 0.09 | 1.14 (0.91-1.43) | 0.25 |
| **Thyroiditis** | **Unexposed** | 220 | 40.61 | Ref. |  | Ref. |  |
|  | **<30 days** | 10 | 2.59 | 1.42 (0.54-3.71) | 0.47 | 1.22 (0.48-3.09) | 0.67 |
|  | **30-180 days ago** | 40 | 8.24 | 1.54 (0.89-2.68) | 0.13 | 1.35 (0.77-2.37) | 0.29 |
| **Hashimoto thyroiditis** | **Unexposed** | 130 | 40.63 | Ref. |  | Ref. |  |
|  | **<30 days** | 5 | 2.59 | 1.08 (0.32-3.67) | 0.90 | 0.85 (0.25-2.83) | 0.79 |
|  | **30-180 days ago** | 25 | 8.24 | 1.60 (0.83-3.07) | 0.16 | 1.26 (0.63-2.53) | 0.51 |
| **Addison’s disease** | **Unexposed** | 48 | 40.65 | Ref. |  | Ref. |  |
|  | **<30 days** | 3 | 2.59 | 0.77 (0.19-3.08) | 0.72 | 0.72 (0.18-2.89) | 0.64 |
|  | **30-180 days ago** | 19 | 8.25 | 2.65 (1.13-6.20) | 0.02 | 2.46 (0.99-6.12) | 0.05 |
| **Gastrointestinal outcomes** | | | | | | | |
| **Crohn’s disease** | **Unexposed** | 1019 | 40.49 | Ref. |  | Ref. |  |
|  | **<30 days** | 99 | 2.58 | 1.90 (1.43-2.53) | 0.00 | 1.92 (1.43-2.58) | 0.00 |
|  | **30-180 days ago** | 254 | 8.21 | 1.45 (1.16-1.81) | 0.00 | 1.51 (1.20-1.90) | 0.00 |
| **Ulcerative colitis** | **Unexposed** | 1715 | 40.42 | Ref. |  | Ref. |  |
|  | **<30 days** | 124 | 2.57 | 1.23 (0.97-1.55) | 0.09 | 1.15 (0.90-1.47) | 0.25 |
|  | **30-180 days ago** | 434 | 8.19 | 1.27 (1.07-1.50) | 0.01 | 1.19 (1.00-1.41) | 0.05 |
| **Celiac Disease** | **Unexposed** | 703 | 40.50 | Ref. |  | Ref. |  |
|  | **<30 days** | 63 | 2.58 | 1.70 (1.23-2.37) | 0.00 | 1.52 (1.08-2.12) | 0.01 |
|  | **30-180 days ago** | 166 | 8.21 | 1.35 (1.05-1.73) | 0.02 | 1.19 (0.91-1.55) | 0.20 |
| **Autoimmune hepatitis** | **Unexposed** | 74 | 40.65 | Ref. |  | Ref. |  |
|  | **<30 days** | 3 | 2.59 | 0.87 (0.21-3.66) | 0.85 | 0.72 (0.16-3.11) | 0.65 |
|  | **30-180 days ago** | 27 | 8.25 | 2.81 (1.14-6.92) | 0.02 | 2.34 (0.86-6.38) | 0.10 |
| **Hematologic outcomes** | | | | | | | |
| **Purpura, thrombocytopenia including Henoch–Schönlein purpura (HSP)** | **Unexposed** | 449 | 40.59 | Ref. |  | Ref. |  |
|  | **<30 days** | 19 | 2.59 | 0.72 (0.40-1.28) | 0.26 | 0.78 (0.43-1.39) | 0.39 |
|  | **30-180 days ago** | 84 | 8.23 | 0.95 (0.64-1.40) | 0.78 | 1.00 (0.66-1.50) | 0.98 |
| **Idiopathic thrombocytopenic purpura (ITP)** | **Unexposed** | 131 | 40.65 | Ref. |  | Ref. |  |
|  | **<30 days** | 4 | 2.59 | 0.35 (0.10-1.26) | 0.11 | 0.36 (0.10-1.28) | 0.12 |
|  | **30-180 days ago** | 30 | 8.25 | 0.90 (0.48-1.72) | 0.76 | 0.90 (0.47-1.74) | 0.76 |
| **Agranulocytosis** | **Unexposed** | 296 | 40.62 | Ref. |  | Ref. |  |
|  | **<30 days** | 18 | 2.59 | 1.23 (0.62-2.43) | 0.55 | 1.40 (0.70-2.82) | 0.34 |
|  | **30-180 days ago** | 82 | 8.24 | 1.55 (0.96-2.49) | 0.07 | 1.85 (1.15-3.00) | 0.01 |
| **Haemolytic anaemia, including Hemolytic uremic syndrome (HUS)** | **Unexposed** | 3 | 40.66 | Ref. |  | Ref. |  |
|  | **<30 days** | 0 | 2.59 | Na | Na | Na | Na |
|  | **30-180 days ago** | 3 | 8.25 | 1.28 (0.23-7.05) | 0.78 | 0.95 (0.13-6.96) | 0.96 |
| **Neurologic outcomes** | | | | | | | |
| **Multiple sclerosis** | **Unexposed** | 895 | 40.58 | Ref. |  | Ref. |  |
|  | **<30 days** | 66 | 2.59 | 1.28 (0.90-1.82) | 0.17 | 1.13 (0.80-1.61) | 0.49 |
|  | **30-180 days ago** | 207 | 8.23 | 1.23 (0.96-1.57) | 0.10 | 1.10 (0.86-1.41) | 0.44 |
| **Demyelinating disease of the central nervous system** | **Unexposed** | 170 | 40.64 | Ref. |  | Ref. |  |
|  | **<30 days** | 11 | 2.59 | 1.44 (0.70-2.95) |  | 1.36 (0.66-2.78) | 0.40 |
|  | **30-180 days ago** | 38 | 8.24 | 1.21 (0.73-2.00) |  | 1.14 (0.68-1.90) | 0.61 |
| **Acute disseminated encephalomyelitis (ADEM)** | **Unexposed** | NA | NA | Ref. |  | Ref. |  |
|  | **<30 days** | NA | NA | Na | NA | Na | NA |
|  | **30-180 days ago** | NA | NA | Na | Na | Na | NA |
| **Bell's palsy** | **Unexposed** | 277 | 40.60 | Ref. |  | Ref. |  |
|  | **<30 days** | 21 | 2.59 | 1.03 (0.56-1.89) | 0.93 | 1.10 (0.60-2.04) | 0.75 |
|  | **30-180 days ago** | 61 | 8.24 | 1.04 (0.70-1.57) | 0.83 | 1.09 (0.73-1.64) | 0.66 |
| **Narcolepsy** | **Unexposed** | 71 | 40.65 | Ref. |  | Ref. |  |
|  | **<30 days** | 1 | 2.59 | 0.12 (0.01-0.99) | 0.05 | 0.09 (0.01-0.73) | 0.02 |
|  | **30-180 days ago** | 12 | 8.25 | 0.82 (0.30-2.22) | 0.70 | 0.62 (0.22-1.73) | 0.36 |
| **Polyneuropathy** | **Unexposed** | 194 | 40.62 | Ref. |  | Ref. |  |
|  | **<30 days** | 14 | 2.59 | 1.73 (0.78-3.84) | 0.18 | 1.83 (0.84-4.02) | 0.13 |
|  | **30-180 days ago** | 37 | 8.24 | 1.71 (0.96-3.06) | 0.07 | 1.76 (0.97-3.18) | 0.06 |
| **Myasthenia gravis** | **Unexposed** | 36 | 40.66 | Ref. |  | Ref. |  |
|  | **<30 days** | 3 | 2.59 | 3.45 (0.78-15.21) | 0.10 | 2.97 (0.65-13.45) | 0.16 |
|  | **30-180 days ago** | 8 | 8.25 | 1.49 (0.42-5.32) | 0.54 | 1.29 (0.35-4.79) | 0.71 |
| **Rheumatologic outcomes** | | | | | | | |
| **Rheumatoid arthritis** | **Unexposed** | 589 | 40.59 | Ref. |  | Ref. |  |
|  | **<30 days** | 32 | 2.59 | 0.95 (0.60-1.50) | 0.82 | 0.84 (0.53-1.34) | 0.47 |
|  | **30-180 days ago** | 144 | 8.23 | 1.28 (0.96-1.69) | 0.09 | 1.17 (0.88-1.56) | 0.28 |
| **Reactive/idiopathic inflammatory arthritis** | **Unexposed** | 172 | 40.63 | Ref. |  | Ref. |  |
|  | **<30 days** | 5 | 2.59 | 0.38 (0.13-1.07) | 0.07 | 0.34 (0.12-1.02) | 0.05 |
|  | **30-180 days ago** | 23 | 8.24 | 0.59 (0.33-1.07) | 0.08 | 0.56 (0.30-1.08) | 0.08 |
| **Arthralgia** | **Unexposed** | 4670 | 39.46 | Ref. |  | Ref. |  |
|  | **<30 days** | 310 | 2.51 | 1.23 (1.06-1.43) | 0.01 | 1.15 (0.99-1.33) | 0.08 |
|  | **30-180 days ago** | 1065 | 7.99 | 1.14 (1.03-1.26) | 0.01 | 1.08 (0.97-1.20) | 0.16 |
| **Connective tissue diseases** | **Unexposed** | 314 | 40.60 | Ref. |  | Ref. |  |
|  | **<30 days** | 22 | 2.59 | 1.26 (0.67-2.37) | 0.47 | 1.15 (0.62-2.12) | 0.65 |
|  | **30-180 days ago** | 67 | 8.24 | 1.11 (0.73-1.67) | 0.63 | 1.03 (0.69-1.55) | 0.88 |
| **Dermatopolymyositis** | **Unexposed** | 29 | 40.66 | Ref. |  | Ref. |  |
|  | **<30 days** | 4 | 2.59 | 1.99 (0.49-8.04) | 0.33 | 1.73 (0.43-7.02) | 0.44 |
|  | **30-180 days ago** | 7 | 8.25 | 0.72 (0.24-2.14) | 0.56 | 0.64 (0.21-1.93) | 0.43 |
| **Sjogren’s syndrome** | **Unexposed** | 124 | 40.63 | Ref. |  | Ref. |  |
|  | **<30 days** | 10 | 2.59 | 0.97 (0.41-2.33) | 0.95 | 0.91 (0.39-2.09) | 0.82 |
|  | **30-180 days ago** | 23 | 8.24 | 0.72 (0.39-1.31) | 0.28 | 0.69 (0.38-1.27) | 0.24 |
| **SLE (Lupus)** | **Unexposed** | 106 | 40.64 | Ref. |  | Ref. |  |
|  | **<30 days** | 5 | 2.59 | 0.96 (0.24-3.73) | 0.95 | 0.87 (0.23-3.33) | 0.84 |
|  | **30-180 days ago** | 24 | 8.24 | 1.55 (0.64-3.78) | 0.34 | 1.37 (0.57-3.32) | 0.48 |
| **Systemic sclerosis** | **Unexposed** | 40 | 40.66 | Ref. |  | Ref. |  |
|  | **<30 days** | 1 | 2.59 | 0.32 (0.04-2.74) | 0.30 | 0.34 (0.04-2.74) | 0.31 |
|  | **30-180 days ago** | 7 | 8.25 | 0.84 (0.38-1.87) | 0.67 | 0.88 (0.41-1.89) | 0.75 |

Supplemental table 7. SARS-CoV-2 infection and autoimmune conditions (all outcomes) among 40–65-year-olds, using a 0-30 and 30-180 days risk window. HR are adjusted for age, sex, health region, municipality size, household size, household crowding, low family income, country of origin.

| **Disease** | **Time from exposure** | **Number of failure** | **Person years at risk** | **Unadjusted** | | **Adjusted** | |
| --- | --- | --- | --- | --- | --- | --- | --- |
|  |  |  |  | **HR (95% CI)** | **p-value** | **HR (95% CI)** | **p-value** |
| **Dermatologic outcomes** | | | | | | | |
| **Urticaria** | **Unexposed** | 214 | 36.60 | Ref. |  | Ref. |  |
|  | **<30 days** | 2 | 0.21 | 1.58 (0.38-6.52) | 0.53 | 1.62 (0.39-6.73) | 0.51 |
|  | **30-180 days ago** | 4 | 0.38 | 1.76 (0.65-4.74) | 0.27 | 1.91 (0.70-5.23) | 0.21 |
| **Erythema multiforme including Stevens–Johnson syndrome** | **Unexposed** | 13 | 36.65 | Ref. |  | Ref. |  |
|  | **<30 days** | 0 | 0.21 | Na | Na | Na | Na |
|  | **30-180 days ago** | 0 | 0.38 | Na | Na | Na | Na |
| **Erythema nodosum** | **Unexposed** | 24 | 36.65 | Ref. |  | Ref. |  |
|  | **<30 days** | 0 | 0.21 | Na | Na | Na | Na |
|  | **30-180 days ago** | 0 | 0.38 | Na | Na | Na | Na |
| **Alopecia** | **Unexposed** | 24 | 36.65 | Ref. |  | Ref. |  |
|  | **<30 days** | 0 | 0.21 | Na | Na | Na | Na |
|  | **30-180 days ago** | 0 | 0.38 | Na | Na | Na | Na |
| **Endocrinologic outcomes** | | | | | | | |
| **Hyperthyroidism** | **Unexposed** | 2165 | 36.43 | Ref. |  | Ref. |  |
|  | **<30 days** | 9 | 0.21 | 0.76 (0.39-1.47) | 0.42 | 0.73 (0.38-1.42) | 0.35 |
|  | **30-180 days ago** | 24 | 0.37 | 1.10 (0.73-1.65) | 0.65 | 1.05 (0.70-1.58) | 0.80 |
| **Graves Disease** | **Unexposed** | 1320 | 36.52 | Ref. |  | Ref. |  |
|  | **<30 days** | 8 | 0.21 | 1.15 (0.57-2.32) | 0.70 | 1.06 (0.53-2.14) | 0.87 |
|  | **30-180 days ago** | 14 | 0.38 | 1.08 (0.63-1.83) | 0.79 | 1.00 (0.58-1.70) | 0.99 |
| **Thyroiditis** | **Unexposed** | 253 | 36.60 | Ref. |  | Ref. |  |
|  | **<30 days** | 0 | 0.21 | Na | Na | Na | Na |
|  | **30-180 days ago** | 0 | 0.38 | Na | Na | Na | Na |
| **Hashimoto thyroiditis** | **Unexposed** | 99 | 36.63 | Ref. |  | Ref. |  |
|  | **<30 days** | 0 | 0.21 | Na | Na | Na | Na |
|  | **30-180 days ago** | 0 | 0.38 | Na | Na | Na | Na |
| **Addison’s disease** | **Unexposed** | 60 | 36.64 | Ref. |  | Ref. |  |
|  | **<30 days** | 0 | 0.21 | Na | Na | Na | Na |
|  | **30-180 days ago** | 1 | 0.38 | 1.53 (0.24-9.65) | 0.65 | 1.97 (0.31-12.56) | 0.47 |
| **Gastrointestinal outcomes** | | | | | | | |
| **Crohn’s disease** | **Unexposed** | 752 | 36.47 | Ref. |  | Ref. |  |
|  | **<30 days** | 2 | 0.21 | 0.47 (0.12-1.86) | 0.28 | 0.52 (0.13-2.05) | 0.35 |
|  | **30-180 days ago** | 4 | 0.37 | 0.53 (0.20-1.43) | 0.21 | 0.61 (0.23-1.64) | 0.33 |
| **Ulcerative colitis** | **Unexposed** | 1139 | 36.36 | Ref. |  | Ref. |  |
|  | **<30 days** | 6 | 0.21 | 1.06 (0.47-2.37) | 0.89 | 1.08 (0.48-2.44) | 0.85 |
|  | **30-180 days ago** | 7 | 0.37 | 0.66 (0.31-1.39) | 0.27 | 0.70 (0.33-1.49) | 0.35 |
| **Celiac Disease** | **Unexposed** | 414 | 36.57 | Ref. |  | Ref. |  |
|  | **<30 days** | 3 | 0.21 | 1.44 (0.45-4.58) | 0.54 | 1.50 (0.47-4.80) | 0.49 |
|  | **30-180 days ago** | 1 | 0.38 | 0.26 (0.04-1.88) | 0.18 | 0.28 (0.04-2.05) | 0.21 |
| **Autoimmune hepatitis** | **Unexposed** | 169 | 36.63 | Ref. |  | Ref. |  |
|  | **<30 days** | 0 | 0.21 | Na | Na | Na | Na |
|  | **30-180 days ago** | 1 | 0.38 | 0.63 (0.08-4.67) | 0.65 | 0.77 (0.10-5.82) | 0.80 |
| **Hematologic outcomes** | | | | | | | |
| **Purpura, thrombocytopenia including Henoch–Schönlein purpura (HSP)** | **Unexposed** | 687 | 36.55 | Ref. |  | Ref. |  |
|  | **<30 days** | 10 | 0.21 | 3.08 (1.61-5.91) | 0.00 | 3.80 (1.98-7.30) | 0.00 |
|  | **30-180 days ago** | 14 | 0.38 | 2.15 (1.25-3.71) | 0.01 | 2.52 (1.46-4.36) | 0.00 |
| **Idiopathic thrombocytopenic purpura (ITP)** | **Unexposed** | 128 | 36.64 | Ref. |  | Ref. |  |
|  | **<30 days** | 2 | 0.21 | 3.43 (0.83-14.16) | 0.09 | 3.92 (0.93-16.54) | 0.06 |
|  | **30-180 days ago** | 2 | 0.38 | 1.64 (0.39-6.85) | 0.50 | 1.83 (0.45-7.47) | 0.40 |
| **Agranulocytosis** | **Unexposed** | 642 | 36.55 | Ref. |  | Ref. |  |
|  | **<30 days** | 9 | 0.21 | 2.62 (1.32-5.21) | 0.01 | 3.52 (1.76-7.04) | 0.00 |
|  | **30-180 days ago** | 13 | 0.38 | 2.06 (1.16-3.65) | 0.01 | 2.67 (1.50-4.74) | 0.00 |
| **Haemolytic anaemia, including Hemolytic uremic syndrome (HUS)** | **Unexposed** | 8 | 36.66 | Ref. |  | Ref. |  |
|  | **<30 days** | 1 | 0.21 | 27.10 (4.88-150.51) | 0.00 | 30.46 (6.22-149.08) | 0.00 |
|  | **30-180 days ago** | 0 | 0.38 | Na | Na | Na | Na |
| **Neurologic outcomes** | | | | | | | |
| **Multiple sclerosis** | **Unexposed** | 680 | 36.47 | Ref. |  | Ref. |  |
|  | **<30 days** | 0 | 0.21 | Na | Na | Na | Na |
|  | **30-180 days ago** | 3 | 0.38 | 0.44 (0.14-1.38) | 0.16 | 0.47 (0.15-1.45) | 0.19 |
| **Demyelinating disease of the central nervous system** | **Unexposed** | 151 | 36.63 | Ref. |  | Ref. |  |
|  | **<30 days** | 1 | 0.21 | 1.03 (0.14-7.48) |  | 0.86 (0.12-6.34) | 0.88 |
|  | **30-180 days ago** | 1 | 0.38 | 0.61 (0.09-4.25) |  | 0.54 (0.08-3.84) | 0.54 |
| **Acute disseminated encephalomyelitis (ADEM)** | **Unexposed** | NA | NA | Ref. |  | Ref. |  |
|  | **<30 days** | NA | NA | Na | NA | Na | NA |
|  | **30-180 days ago** | NA | NA | Na | Na | Na | NA |
| **Bell's palsy** | **Unexposed** | 391 | 36.58 | Ref. |  | Ref. |  |
|  | **<30 days** | 3 | 0.21 | 1.18 (0.38-3.66) | 0.78 | 1.20 (0.39-3.70) | 0.75 |
|  | **30-180 days ago** | 6 | 0.38 | 1.33 (0.59-3.02) | 0.49 | 1.32 (0.58-2.99) | 0.51 |
| **Narcolepsy** | **Unexposed** | 23 | 36.65 | Ref. |  | Ref. |  |
|  | **<30 days** | 0 | 0.21 | Na | Na | Na | Na |
|  | **30-180 days ago** | 0 | 0.38 | Na | Na | Na | Na |
| **Polyneuropathy** | **Unexposed** | 899 | 36.42 | Ref. |  | Ref. |  |
|  | **<30 days** | 6 | 0.21 | 1.37 (0.61-3.08) | 0.45 | 1.81 (0.80-4.08) | 0.15 |
|  | **30-180 days ago** | 15 | 0.37 | 1.87 (1.11-3.16) | 0.02 | 2.42 (1.43-4.09) | 0.00 |
| **Myasthenia gravis** | **Unexposed** | 76 | 36.65 | Ref. |  | Ref. |  |
|  | **<30 days** | 0 | 0.21 | Na | Na | Na | Na |
|  | **30-180 days ago** | 1 | 0.38 | 1.09 (0.14-8.48) | 0.93 | 1.26 (0.17-9.64) | 0.82 |
| **Rheumatologic outcomes** | | | | | | | |
| **Rheumatoid arthritis** | **Unexposed** | 1434 | 36.38 | Ref. |  | Ref. |  |
|  | **<30 days** | 5 | 0.21 | 0.65 (0.27-1.58) | 0.34 | 0.79 (0.32-1.92) | 0.60 |
|  | **30-180 days ago** | 9 | 0.37 | 0.64 (0.33-1.23) | 0.18 | 0.76 (0.40-1.47) | 0.42 |
| **Reactive/idiopathic inflammatory arthritis** | **Unexposed** | 99 | 36.63 | Ref. |  | Ref. |  |
|  | **<30 days** | 4 | 0.21 | 8.39 (2.73-25.81) | 0.00 | 8.93 (2.80-28.46) | 0.00 |
|  | **30-180 days ago** | 1 | 0.38 | 1.07 (0.15-7.50) | 0.95 | 1.23 (0.17-8.74) | 0.84 |
| **Arthralgia** | **Unexposed** | 5521 | 35.09 | Ref. |  | Ref. |  |
|  | **<30 days** | 23 | 0.20 | 0.67 (0.44-1.01) | 0.06 | 0.62 (0.41-0.94) | 0.02 |
|  | **30-180 days ago** | 68 | 0.36 | 1.11 (0.87-1.41) | 0.41 | 0.99 (0.77-1.26) | 0.91 |
| **Connective tissue diseases** | **Unexposed** | 519 | 36.49 | Ref. |  | Ref. |  |
|  | **<30 days** | 2 | 0.21 | 0.81 (0.20-3.32) | 0.77 | 0.90 (0.22-3.68) | 0.89 |
|  | **30-180 days ago** | 8 | 0.38 | 1.70 (0.85-3.40) | 0.14 | 1.88 (0.94-3.79) | 0.08 |
| **Dermatopolymyositis** | **Unexposed** | 51 | 36.65 | Ref. |  | Ref. |  |
|  | **<30 days** | 1 | 0.21 | 2.58 (0.29-23.32) | 0.40 | 2.94 (0.34-25.23) | 0.32 |
|  | **30-180 days ago** | 0 | 0.38 | Na | Na | Na | Na |
| **Sjogren’s syndrome** | **Unexposed** | 285 | 36.55 | Ref. |  | Ref. |  |
|  | **<30 days** | 1 | 0.21 | 0.76 (0.11-5.41) | 0.78 | 0.89 (0.12-6.33) | 0.90 |
|  | **30-180 days ago** | 6 | 0.38 | 2.36 (1.05-5.27) | 0.04 | 2.75 (1.23-6.16) | 0.01 |
| **SLE (Lupus)** | **Unexposed** | 93 | 36.62 | Ref. |  | Ref. |  |
|  | **<30 days** | 0 | 0.21 | Na | Na | Na | Na |
|  | **30-180 days ago** | 2 | 0.38 | 2.63 (0.67-10.30) | 0.16 | 2.29 (0.57-9.23) | 0.24 |
| **Systemic sclerosis** | **Unexposed** | 119 | 36.64 | Ref. |  | Ref. |  |
|  | **<30 days** | 0 | 0.21 | Na | Na | Na | Na |
|  | **30-180 days ago** | 0 | 0.38 | Na | Na | Na | Na |

Supplemental table 8. Vaccination and autoimmune conditions (all outcomes) among 40–65-year-olds, using a 0-30 and 30-180 days risk window. HR are adjusted for age, sex, health region, municipality size, household size, household crowding, low family income, country of origin, and previous covid infection.

| **Disease** | **Time from exposure** | **Number of failure** | **Person years at risk** | **Unadjusted** | | **Adjusted** | |
| --- | --- | --- | --- | --- | --- | --- | --- |
|  |  |  |  | **HR (95% CI)** | **p-value** | **HR (95% CI)** | **p-value** |
| **Dermatologic outcomes** | | | | | | | |
| **Urticaria** | **Unexposed** | 277 | 43.42 | Ref. |  | Ref. |  |
|  | **<30 days** | 17 | 3.46 | 0.83 (0.43-1.62) | 0.59 | 0.86 (0.45-1.65) | 0.65 |
|  | **30-180 days ago** | 76 | 11.37 | 1.11 (0.70-1.74) | 0.66 | 1.13 (0.72-1.78) | 0.59 |
| **Erythema multiforme including Stevens–Johnson syndrome** | **Unexposed** | 16 | 43.48 | Ref. |  | Ref. |  |
|  | **<30 days** | 3 | 3.47 | 4.06 (0.52-31.71) | 0.18 | 3.50 (0.42-29.24) | 0.25 |
|  | **30-180 days ago** | 4 | 11.38 | 0.91 (0.10-8.53) | 0.93 | 0.72 (0.06-8.13) | 0.79 |
| **Erythema nodosum** | **Unexposed** | 17 | 43.48 | Ref. |  | Ref. |  |
|  | **<30 days** | 7 | 3.47 | 6.33 (1.93-20.76) | 0.00 | 5.93 (1.89-18.55) | 0.00 |
|  | **30-180 days ago** | 9 | 11.38 | 4.47 (1.63-12.22) | 0.00 | 4.29 (1.67-10.98) | 0.00 |
| **Alopecia** | **Unexposed** | 29 | 43.48 | Ref. |  | Ref. |  |
|  | **<30 days** | 2 | 3.47 | 0.77 (0.09-6.84) | 0.82 | 0.61 (0.07-4.94) | 0.64 |
|  | **30-180 days ago** | 9 | 11.38 | 0.86 (0.23-3.15) | 0.81 | 0.66 (0.17-2.53) | 0.55 |
| **Endocrinologic outcomes** | | | | | | | |
| **Hyperthyroidism** | **Unexposed** | 2416 | 43.21 | Ref. |  | Ref. |  |
|  | **<30 days** | 211 | 3.44 | 1.16 (0.96-1.40) | 0.14 | 1.10 (0.91-1.33) | 0.35 |
|  | **30-180 days ago** | 661 | 11.31 | 1.09 (0.93-1.26) | 0.29 | 1.03 (0.89-1.20) | 0.68 |
| **Graves Disease** | **Unexposed** | 1475 | 43.32 | Ref. |  | Ref. |  |
|  | **<30 days** | 127 | 3.45 | 1.05 (0.83-1.33) | 0.68 | 1.03 (0.81-1.30) | 0.82 |
|  | **30-180 days ago** | 377 | 11.34 | 0.98 (0.81-1.19) | 0.86 | 0.96 (0.79-1.16) | 0.65 |
| **Thyroiditis** | **Unexposed** | 309 | 43.42 | Ref. |  | Ref. |  |
|  | **<30 days** | 25 | 3.46 | 1.49 (0.80-2.76) | 0.21 | 1.45 (0.78-2.70) | 0.24 |
|  | **30-180 days ago** | 64 | 11.36 | 0.92 (0.56-1.52) | 0.75 | 0.91 (0.55-1.50) | 0.71 |
| **Hashimoto thyroiditis** | **Unexposed** | 127 | 43.46 | Ref. |  | Ref. |  |
|  | **<30 days** | 7 | 3.47 | 1.52 (0.47-4.88) | 0.49 | 1.32 (0.42-4.13) | 0.64 |
|  | **30-180 days ago** | 23 | 11.38 | 0.94 (0.46-1.93) | 0.87 | 0.85 (0.42-1.72) | 0.65 |
| **Addison’s disease** | **Unexposed** | 55 | 43.47 | Ref. |  | Ref. |  |
|  | **<30 days** | 3 | 3.47 | 0.43 (0.12-1.59) | 0.21 | 0.37 (0.10-1.40) | 0.14 |
|  | **30-180 days ago** | 17 | 11.38 | 0.77 (0.31-1.91) | 0.58 | 0.65 (0.25-1.71) | 0.38 |
| **Gastrointestinal outcomes** | | | | | | | |
| **Crohn’s disease** | **Unexposed** | 870 | 43.28 | Ref. |  | Ref. |  |
|  | **<30 days** | 72 | 3.45 | 1.36 (0.99-1.86) | 0.06 | 1.28 (0.93-1.78) | 0.13 |
|  | **30-180 days ago** | 226 | 11.32 | 1.14 (0.89-1.47) | 0.29 | 1.06 (0.82-1.38) | 0.66 |
| **Ulcerative colitis** | **Unexposed** | 1256 | 43.16 | Ref. |  | Ref. |  |
|  | **<30 days** | 106 | 3.44 | 1.36 (1.04-1.77) | 0.03 | 1.37 (1.04-1.80) | 0.02 |
|  | **30-180 days ago** | 369 | 11.29 | 1.55 (1.26-1.91) | 0.00 | 1.58 (1.27-1.96) | 0.00 |
| **Celiac Disease** | **Unexposed** | 468 | 43.38 | Ref. |  | Ref. |  |
|  | **<30 days** | 35 | 3.46 | 1.37 (0.85-2.21) | 0.20 | 1.29 (0.80-2.09) | 0.30 |
|  | **30-180 days ago** | 142 | 11.35 | 1.77 (1.26-2.47) | 0.00 | 1.63 (1.16-2.31) | 0.01 |
| **Autoimmune hepatitis** | **Unexposed** | 188 | 43.46 | Ref. |  | Ref. |  |
|  | **<30 days** | 10 | 3.47 | 0.61 (0.28-1.35) | 0.22 | 0.49 (0.21-1.10) | 0.08 |
|  | **30-180 days ago** | 55 | 11.38 | 1.19 (0.73-1.95) | 0.47 | 0.94 (0.56-1.56) | 0.80 |
| **Hematologic outcomes** | | | | | | | |
| **Purpura, thrombocytopenia including Henoch–Schönlein purpura (HSP)** | **Unexposed** | 781 | 43.38 | Ref. |  | Ref. |  |
|  | **<30 days** | 54 | 3.46 | 0.82 (0.56-1.21) | 0.33 | 0.82 (0.56-1.22) | 0.33 |
|  | **30-180 days ago** | 205 | 11.35 | 1.13 (0.83-1.53) | 0.43 | 1.13 (0.84-1.53) | 0.42 |
| **Idiopathic thrombocytopenic purpura (ITP)** | **Unexposed** | 153 | 43.47 | Ref. |  | Ref. |  |
|  | **<30 days** | 13 | 3.47 | 0.99 (0.45-2.18) | 0.98 | 0.98 (0.44-2.16) | 0.96 |
|  | **30-180 days ago** | 39 | 11.38 | 1.14 (0.58-2.25) | 0.71 | 1.12 (0.57-2.19) | 0.75 |
| **Agranulocytosis** | **Unexposed** | 739 | 43.38 | Ref. |  | Ref. |  |
|  | **<30 days** | 61 | 3.46 | 0.99 (0.68-1.44) | 0.96 | 1.01 (0.69-1.48) | 0.95 |
|  | **30-180 days ago** | 203 | 11.35 | 1.06 (0.78-1.42) | 0.72 | 1.05 (0.77-1.43) | 0.76 |
| **Haemolytic anaemia, including Hemolytic uremic syndrome (HUS)** | **Unexposed** | 9 | 43.49 | Ref. |  | Ref. |  |
|  | **<30 days** | 0 | 3.47 | Na | Na | Na | Na |
|  | **30-180 days ago** | 5 | 11.38 | 0.41 (0.10-1.69) | 0.22 | 0.46 (0.11-1.90) | 0.28 |
| **Neurologic outcomes** | | | | | | | |
| **Multiple sclerosis** | **Unexposed** | 794 | 43.29 | Ref. |  | Ref. |  |
|  | **<30 days** | 59 | 3.45 | 1.09 (0.77-1.55) | 0.63 | 1.09 (0.75-1.57) | 0.65 |
|  | **30-180 days ago** | 208 | 11.32 | 1.20 (0.92-1.57) | 0.18 | 1.16 (0.88-1.53) | 0.29 |
| **Demyelinating disease of the central nervous system** | **Unexposed** | 180 | 43.46 | Ref. |  | Ref. |  |
|  | **<30 days** | 13 | 3.46 | 1.10 (0.46-2.64) |  | 1.05 (0.44-2.51) | 0.92 |
|  | **30-180 days ago** | 50 | 11.37 | 1.07 (0.59-1.97) |  | 1.03 (0.56-1.91) | 0.92 |
| **Acute disseminated encephalomyelitis (ADEM)** | **Unexposed** | NA | NA | Ref. |  | Ref. |  |
|  | **<30 days** | NA | NA | Na | NA | Na | NA |
|  | **30-180 days ago** | NA | NA | Na | Na | Na | NA |
| **Bell's palsy** | **Unexposed** | 414 | 43.40 | Ref. |  | Ref. |  |
|  | **<30 days** | 48 | 3.46 | 1.54 (0.99-2.39) | 0.06 | 1.49 (0.95-2.33) | 0.09 |
|  | **30-180 days ago** | 135 | 11.36 | 1.24 (0.87-1.77) | 0.23 | 1.22 (0.85-1.76) | 0.28 |
| **Narcolepsy** | **Unexposed** | 18 | 43.48 | Ref. |  | Ref. |  |
|  | **<30 days** | 3 | 3.47 | 2.43 (0.37-16.13) | 0.36 | 2.73 (0.39-19.13) | 0.31 |
|  | **30-180 days ago** | 9 | 11.38 | 1.57 (0.37-6.63) | 0.54 | 1.80 (0.38-8.58) | 0.46 |
| **Polyneuropathy** | **Unexposed** | 1010 | 43.23 | Ref. |  | Ref. |  |
|  | **<30 days** | 87 | 3.44 | 1.26 (0.93-1.70) | 0.13 | 1.15 (0.85-1.57) | 0.35 |
|  | **30-180 days ago** | 259 | 11.31 | 1.21 (0.95-1.55) | 0.12 | 1.12 (0.86-1.44) | 0.40 |
| **Myasthenia gravis** | **Unexposed** | 68 | 43.48 | Ref. |  | Ref. |  |
|  | **<30 days** | 5 | 3.47 | 0.56 (0.18-1.77) | 0.32 | 0.56 (0.18-1.75) | 0.32 |
|  | **30-180 days ago** | 36 | 11.38 | 1.74 (0.75-4.04) | 0.20 | 1.71 (0.77-3.81) | 0.19 |
| **Rheumatologic outcomes** | | | | | | | |
| **Rheumatoid arthritis** | **Unexposed** | 1617 | 43.21 | Ref. |  | Ref. |  |
|  | **<30 days** | 140 | 3.44 | 1.33 (1.04-1.70) | 0.02 | 1.15 (0.90-1.48) | 0.27 |
|  | **30-180 days ago** | 418 | 11.30 | 1.31 (1.08-1.60) | 0.01 | 1.12 (0.91-1.38) | 0.27 |
| **Reactive/idiopathic inflammatory arthritis** | **Unexposed** | 129 | 43.46 | Ref. |  | Ref. |  |
|  | **<30 days** | 8 | 3.46 | 0.61 (0.22-1.72) | 0.35 | 0.60 (0.20-1.75) | 0.35 |
|  | **30-180 days ago** | 30 | 11.37 | 0.97 (0.44-2.14) | 0.94 | 0.93 (0.40-2.16) | 0.87 |
| **Arthralgia** | **Unexposed** | 6712 | 41.64 | Ref. |  | Ref. |  |
|  | **<30 days** | 517 | 3.31 | 1.08 (0.96-1.22) | 0.18 | 1.03 (0.92-1.16) | 0.59 |
|  | **30-180 days ago** | 1952 | 10.87 | 1.26 (1.15-1.37) | 0.00 | 1.21 (1.10-1.32) | 0.00 |
| **Connective tissue diseases** | **Unexposed** | 603 | 43.31 | Ref. |  | Ref. |  |
|  | **<30 days** | 52 | 3.45 | 1.19 (0.81-1.77) | 0.38 | 1.07 (0.73-1.58) | 0.72 |
|  | **30-180 days ago** | 136 | 11.33 | 0.90 (0.66-1.23) | 0.52 | 0.79 (0.58-1.08) | 0.14 |
| **Dermatopolymyositis** | **Unexposed** | 66 | 43.48 | Ref. |  | Ref. |  |
|  | **<30 days** | 3 | 3.47 | 0.31 (0.07-1.41) | 0.13 | 0.26 (0.06-1.19) | 0.08 |
|  | **30-180 days ago** | 15 | 11.38 | 0.50 (0.21-1.18) | 0.11 | 0.38 (0.16-0.89) | 0.03 |
| **Sjogren’s syndrome** | **Unexposed** | 327 | 43.37 | Ref. |  | Ref. |  |
|  | **<30 days** | 28 | 3.46 | 1.32 (0.78-2.23) | 0.29 | 1.22 (0.73-2.04) | 0.45 |
|  | **30-180 days ago** | 76 | 11.35 | 1.10 (0.73-1.65) | 0.64 | 1.03 (0.69-1.53) | 0.90 |
| **SLE (Lupus)** | **Unexposed** | 107 | 43.45 | Ref. |  | Ref. |  |
|  | **<30 days** | 11 | 3.46 | 1.51 (0.56-4.09) | 0.42 | 1.50 (0.56-4.00) | 0.42 |
|  | **30-180 days ago** | 22 | 11.37 | 0.97 (0.44-2.11) | 0.94 | 0.95 (0.42-2.12) | 0.89 |
| **Systemic sclerosis** | **Unexposed** | 134 | 43.47 | Ref. |  | Ref. |  |
|  | **<30 days** | 12 | 3.47 | 1.62 (0.73-3.63) | 0.24 | 1.30 (0.59-2.88) | 0.51 |
|  | **30-180 days ago** | 28 | 11.38 | 0.93 (0.44-2.00) | 0.86 | 0.65 (0.31-1.37) | 0.26 |

Supplemental table 9. SARS-CoV-2 infection and autoimmune conditions (all outcomes) among 18–65-year-olds, using a 0-30 and 30-365 days risk window. HR are adjusted for age, sex, health region, municipality size, household size, household crowding, low family income, country of origin.

| **Disease** | **Time from exposure** | **Number of failure** | **Person years at risk** | **Unadjusted** | | **Adjusted** | |
| --- | --- | --- | --- | --- | --- | --- | --- |
|  |  |  |  | **HR (95% CI)** | **p-value** | **HR (95% CI)** | **p-value** |
| **Dermatologic outcomes** | | | | | | | |
| **Urticaria** | **Unexposed** | 440 | 67.53 | Ref. |  | Ref. |  |
|  | **<30 days** | 3 | 0.51 | 0.75 (0.24-2.37) | 0.63 | 0.76 (0.24-2.39) | 0.63 |
|  | **30-365 days ago** | 12 | 1.31 | 1.20 (0.67-2.15) | 0.54 | 1.31 (0.73-2.36) | 0.37 |
| **Erythema multiforme including Stevens–Johnson syndrome** | **Unexposed** | 24 | 67.63 | Ref. |  | Ref. |  |
|  | **<30 days** | 1 | 0.51 | 6.54 (0.93-46.15) | 0.06 | 6.96 (0.94-51.55) | 0.06 |
|  | **30-365 days ago** | 2 | 1.31 | 4.67 (1.02-21.32) | 0.05 | 5.34 (1.07-26.53) | 0.04 |
| **Erythema nodosum** | **Unexposed** | 53 | 67.62 | Ref. |  | Ref. |  |
|  | **<30 days** | 0 | 0.51 | Na | Na | Na | Na |
|  | **30-365 days ago** | 1 | 1.31 | 0.80 (0.11-5.64) | 0.82 | 0.86 (0.12-6.34) | 0.88 |
| **Alopecia** | **Unexposed** | 56 | 67.61 | Ref. |  | Ref. |  |
|  | **<30 days** | 0 | 0.51 | Na | Na | Na | Na |
|  | **30-365 days ago** | 0 | 1.31 | Na | Na | Na | Na |
| **Endocrinologic outcomes** | | | | | | | |
| **Hyperthyroidism** | **Unexposed** | 3469 | 67.29 | Ref. |  | Ref. |  |
|  | **<30 days** | 18 | 0.51 | 0.72 (0.45-1.15) | 0.17 | 0.73 (0.45-1.16) | 0.18 |
|  | **30-365 days ago** | 74 | 1.30 | 1.13 (0.89-1.42) | 0.32 | 1.16 (0.92-1.46) | 0.22 |
| **Graves Disease** | **Unexposed** | 2223 | 67.42 | Ref. |  | Ref. |  |
|  | **<30 days** | 15 | 0.51 | 0.95 (0.57-1.59) | 0.84 | 0.92 (0.55-1.55) | 0.76 |
|  | **30-365 days ago** | 50 | 1.31 | 1.19 (0.90-1.58) | 0.23 | 1.18 (0.89-1.57) | 0.25 |
| **Thyroiditis** | **Unexposed** | 422 | 67.54 | Ref. |  | Ref. |  |
|  | **<30 days** | 0 | 0.51 | Na | Na | Na | Na |
|  | **30-365 days ago** | 4 | 1.31 | 0.58 (0.22-1.56) | 0.28 | 0.53 (0.20-1.42) | 0.21 |
| **Hashimoto thyroiditis** | **Unexposed** | 197 | 67.59 | Ref. |  | Ref. |  |
|  | **<30 days** | 0 | 0.51 | Na | Na | Na | Na |
|  | **30-365 days ago** | 2 | 1.31 | 0.70 (0.18-2.72) | 0.60 | 0.60 (0.15-2.35) | 0.46 |
| **Addison’s disease** | **Unexposed** | 100 | 67.61 | Ref. |  | Ref. |  |
|  | **<30 days** | 1 | 0.51 | 0.86 (0.12-6.13) | 0.88 | 1.01 (0.14-7.24) | 0.99 |
|  | **30-365 days ago** | 2 | 1.31 | 0.78 (0.19-3.19) | 0.73 | 0.94 (0.22-3.97) | 0.94 |
| **Gastrointestinal outcomes** | | | | | | | |
| **Crohn’s disease** | **Unexposed** | 1578 | 67.31 | Ref. |  | Ref. |  |
|  | **<30 days** | 10 | 0.51 | 0.79 (0.43-1.49) | 0.47 | 0.80 (0.43-1.49) | 0.48 |
|  | **30-365 days ago** | 26 | 1.31 | 0.83 (0.56-1.23) | 0.35 | 0.86 (0.58-1.28) | 0.46 |
| **Ulcerative colitis** | **Unexposed** | 2539 | 67.15 | Ref. |  | Ref. |  |
|  | **<30 days** | 21 | 0.51 | 1.12 (0.73-1.74) | 0.60 | 1.13 (0.73-1.76) | 0.59 |
|  | **30-365 days ago** | 45 | 1.30 | 0.91 (0.68-1.23) | 0.56 | 0.97 (0.72-1.30) | 0.82 |
| **Celiac Disease** | **Unexposed** | 973 | 67.43 | Ref. |  | Ref. |  |
|  | **<30 days** | 11 | 0.51 | 1.47 (0.80-2.70) | 0.21 | 1.44 (0.78-2.65) | 0.24 |
|  | **30-365 days ago** | 19 | 1.31 | 0.97 (0.61-1.55) | 0.91 | 1.04 (0.65-1.65) | 0.88 |
| **Autoimmune hepatitis** | **Unexposed** | 237 | 67.60 | Ref. |  | Ref. |  |
|  | **<30 days** | 0 | 0.51 | Na | Na | Na | Na |
|  | **30-365 days ago** | 3 | 1.31 | 0.66 (0.21-2.09) | 0.48 | 0.82 (0.26-2.64) | 0.74 |
| **Hematologic outcomes** | | | | | | | |
| **Purpura, thrombocytopenia including Henoch–Schönlein purpura (HSP)** | **Unexposed** | 1018 | 67.48 | Ref. |  | Ref. |  |
|  | **<30 days** | 15 | 0.51 | 2.29 (1.34-3.92) | 0.00 | 2.77 (1.62-4.76) | 0.00 |
|  | **30-365 days ago** | 30 | 1.31 | 1.65 (1.14-2.39) | 0.01 | 1.92 (1.31-2.80) | 0.00 |
| **Idiopathic thrombocytopenic purpura (ITP)** | **Unexposed** | 226 | 67.61 | Ref. |  | Ref. |  |
|  | **<30 days** | 4 | 0.51 | 2.74 (0.97-7.80) | 0.06 | 2.84 (0.99-8.19) | 0.05 |
|  | **30-365 days ago** | 7 | 1.31 | 1.69 (0.78-3.67) | 0.18 | 1.71 (0.78-3.75) | 0.18 |
| **Agranulocytosis** | **Unexposed** | 866 | 67.49 | Ref. |  | Ref. |  |
|  | **<30 days** | 12 | 0.51 | 1.94 (1.06-3.54) | 0.03 | 2.62 (1.43-4.79) | 0.00 |
|  | **30-365 days ago** | 27 | 1.31 | 1.68 (1.13-2.49) | 0.01 | 2.16 (1.45-3.24) | 0.00 |
| **Haemolytic anaemia, including Hemolytic uremic syndrome (HUS)** | **Unexposed** | 13 | 67.64 | Ref. |  | Ref. |  |
|  | **<30 days** | 1 | 0.51 | 12.88 (2.12-78.20) | 0.01 | 16.51 (2.89-94.30) | 0.00 |
|  | **30-365 days ago** | 0 | 1.31 | Na | Na | Na | Na |
| **Neurologic outcomes** | | | | | | | |
| **Multiple sclerosis** | **Unexposed** | 1415 | 67.38 | Ref. |  | Ref. |  |
|  | **<30 days** | 8 | 0.51 | 0.76 (0.38-1.53) | 0.45 | 0.76 (0.37-1.53) | 0.44 |
|  | **30-365 days ago** | 20 | 1.31 | 0.73 (0.47-1.14) | 0.17 | 0.83 (0.53-1.29) | 0.40 |
| **Demyelinating disease of the central nervous system** | **Unexposed** | 264 | 67.59 | Ref. |  | Ref. |  |
|  | **<30 days** | 2 | 0.51 | 0.84 (0.21-3.40) |  | 0.80 (0.20-3.24) | 0.75 |
|  | **30-365 days ago** | 4 | 1.31 | 0.71 (0.27-1.85) |  | 0.71 (0.27-1.86) | 0.48 |
| **Acute disseminated encephalomyelitis (ADEM)** | **Unexposed** | NA | NA | Ref. |  | Ref. |  |
|  | **<30 days** | NA | NA | Na | NA | Na | NA |
|  | **30-365 days ago** | NA | NA | Na | Na | Na | NA |
| **Bell's palsy** | **Unexposed** | 599 | 67.51 | Ref. |  | Ref. |  |
|  | **<30 days** | 9 | 0.51 | 1.84 (0.95-3.57) | 0.07 | 1.98 (1.02-3.84) | 0.04 |
|  | **30-365 days ago** | 15 | 1.31 | 1.20 (0.72-2.00) | 0.49 | 1.25 (0.74-2.09) | 0.40 |
| **Narcolepsy** | **Unexposed** | 81 | 67.62 | Ref. |  | Ref. |  |
|  | **<30 days** | 0 | 0.51 | Na | Na | Na | Na |
|  | **30-365 days ago** | 0 | 1.31 | Na | Na | Na | Na |
| **Polyneuropathy** | **Unexposed** | 1046 | 67.36 | Ref. |  | Ref. |  |
|  | **<30 days** | 7 | 0.51 | 1.04 (0.49-2.22) | 0.92 | 1.62 (0.76-3.44) | 0.21 |
|  | **30-365 days ago** | 22 | 1.31 | 1.22 (0.79-1.88) | 0.37 | 1.82 (1.18-2.80) | 0.01 |
| **Myasthenia gravis** | **Unexposed** | 103 | 67.62 | Ref. |  | Ref. |  |
|  | **<30 days** | 0 | 0.51 | Na | Na | Na | Na |
|  | **30-365 days ago** | 1 | 1.31 | 0.42 (0.06-3.12) | 0.40 | 0.49 (0.07-3.61) | 0.48 |
| **Rheumatologic outcomes** | | | | | | | |
| **Rheumatoid arthritis** | **Unexposed** | 1884 | 67.31 | Ref. |  | Ref. |  |
|  | **<30 days** | 8 | 0.51 | 0.61 (0.30-1.23) | 0.17 | 0.80 (0.40-1.63) | 0.55 |
|  | **30-365 days ago** | 26 | 1.31 | 0.75 (0.51-1.10) | 0.14 | 0.99 (0.67-1.47) | 0.97 |
| **Reactive/idiopathic inflammatory arthritis** | **Unexposed** | 210 | 67.59 | Ref. |  | Ref. |  |
|  | **<30 days** | 4 | 0.51 | 3.21 (1.13-9.08) | 0.03 | 3.33 (1.15-9.59) | 0.03 |
|  | **30-365 days ago** | 7 | 1.31 | 2.02 (0.92-4.45) | 0.08 | 2.25 (1.01-5.01) | 0.05 |
| **Arthralgia** | **Unexposed** | 8997 | 65.17 | Ref. |  | Ref. |  |
|  | **<30 days** | 60 | 0.49 | 0.79 (0.61-1.02) | 0.07 | 0.78 (0.60-1.01) | 0.06 |
|  | **30-365 days ago** | 218 | 1.26 | 1.15 (1.00-1.32) | 0.05 | 1.12 (0.98-1.29) | 0.10 |
| **Connective tissue diseases** | **Unexposed** | 775 | 67.42 | Ref. |  | Ref. |  |
|  | **<30 days** | 2 | 0.51 | 0.37 (0.09-1.50) | 0.16 | 0.42 (0.10-1.70) | 0.22 |
|  | **30-365 days ago** | 12 | 1.31 | 0.82 (0.46-1.46) | 0.50 | 0.93 (0.52-1.67) | 0.81 |
| **Dermatopolymyositis** | **Unexposed** | 78 | 67.63 | Ref. |  | Ref. |  |
|  | **<30 days** | 1 | 0.51 | 1.29 (0.15-11.04) | 0.82 | 1.45 (0.17-12.21) | 0.73 |
|  | **30-365 days ago** | 0 | 1.31 | Na | Na | Na | Na |
| **Sjogren’s syndrome** | **Unexposed** | 379 | 67.50 | Ref. |  | Ref. |  |
|  | **<30 days** | 1 | 0.51 | 0.37 (0.05-2.65) | 0.32 | 0.46 (0.06-3.33) | 0.44 |
|  | **30-365 days ago** | 10 | 1.31 | 1.40 (0.73-2.66) | 0.31 | 1.76 (0.92-3.37) | 0.09 |
| **SLE (Lupus)** | **Unexposed** | 185 | 67.58 | Ref. |  | Ref. |  |
|  | **<30 days** | 0 | 0.51 | Na | Na | Na | Na |
|  | **30-365 days ago** | 2 | 1.31 | 0.67 (0.17-2.66) | 0.57 | 0.64 (0.16-2.57) | 0.53 |
| **Systemic sclerosis** | **Unexposed** | 147 | 67.62 | Ref. |  | Ref. |  |
|  | **<30 days** | 0 | 0.51 | Na | Na | Na | Na |
|  | **30-365 days ago** | 0 | 1.31 | Na | Na | Na | Na |

Supplemental table 10. Vaccination and autoimmune conditions (all outcomes) among 18–65-year-olds, using a 0-30 and 30-365 days risk window. HR are adjusted for age, sex, health region, municipality size, household size, household crowding, low family income, country of origin, and previous covid infection.

| **Disease** | **Time from exposure** | **Number of failure** | **Person years at risk** | **Unadjusted** | | **Adjusted** | |
| --- | --- | --- | --- | --- | --- | --- | --- |
|  |  |  |  | **HR (95% CI)** | **p-value** | **HR (95% CI)** | **p-value** |
| **Dermatologic outcomes** | | | | | | | |
| **Urticaria** | **Unexposed** | 347 | 55.96 | Ref. |  | Ref. |  |
|  | **<30 days** | 38 | 6.05 | 0.96 (0.64-1.45) | 0.86 | 1.00 (0.66-1.51) | 1.00 |
|  | **30-365 days ago** | 263 | 33.52 | 1.14 (0.86-1.51) | 0.37 | 1.18 (0.88-1.59) | 0.27 |
| **Erythema multiforme including Stevens–Johnson syndrome** | **Unexposed** | 22 | 56.04 | Ref. |  | Ref. |  |
|  | **<30 days** | 4 | 6.05 | 1.57 (0.44-5.51) | 0.49 | 1.49 (0.44-5.09) | 0.52 |
|  | **30-365 days ago** | 15 | 33.57 | 0.78 (0.34-1.80) | 0.56 | 0.71 (0.29-1.76) | 0.46 |
| **Erythema nodosum** | **Unexposed** | 35 | 56.04 | Ref. |  | Ref. |  |
|  | **<30 days** | 14 | 6.05 | 4.32 (1.69-11.03) | 0.00 | 4.04 (1.54-10.61) | 0.00 |
|  | **30-365 days ago** | 27 | 33.57 | 1.84 (0.70-4.89) | 0.22 | 1.78 (0.67-4.74) | 0.25 |
| **Alopecia** | **Unexposed** | 43 | 56.03 | Ref. |  | Ref. |  |
|  | **<30 days** | 6 | 6.05 | 0.91 (0.26-3.10) | 0.88 | 0.86 (0.25-2.91) | 0.81 |
|  | **30-365 days ago** | 27 | 33.57 | 0.66 (0.30-1.47) | 0.31 | 0.64 (0.27-1.49) | 0.30 |
| **Endocrinologic outcomes** | | | | | | | |
| **Hyperthyroidism** | **Unexposed** | 2798 | 55.77 | Ref. |  | Ref. |  |
|  | **<30 days** | 301 | 6.02 | 1.12 (0.96-1.30) | 0.16 | 0.97 (0.83-1.13) | 0.65 |
|  | **30-365 days ago** | 1636 | 33.39 | 1.19 (1.06-1.33) | 0.00 | 1.01 (0.90-1.13) | 0.83 |
| **Graves Disease** | **Unexposed** | 1802 | 55.87 | Ref. |  | Ref. |  |
|  | **<30 days** | 195 | 6.03 | 1.08 (0.89-1.31) | 0.43 | 0.97 (0.80-1.17) | 0.74 |
|  | **30-365 days ago** | 1018 | 33.46 | 1.15 (1.00-1.32) | 0.05 | 1.00 (0.87-1.15) | 0.96 |
| **Thyroiditis** | **Unexposed** | 352 | 55.97 | Ref. |  | Ref. |  |
|  | **<30 days** | 34 | 6.05 | 1.58 (0.95-2.65) | 0.08 | 1.36 (0.82-2.26) | 0.24 |
|  | **30-365 days ago** | 192 | 33.53 | 1.34 (0.92-1.95) | 0.13 | 1.13 (0.78-1.64) | 0.53 |
| **Hashimoto thyroiditis** | **Unexposed** | 179 | 56.01 | Ref. |  | Ref. |  |
|  | **<30 days** | 12 | 6.05 | 1.08 (0.47-2.49) | 0.86 | 0.87 (0.38-1.99) | 0.73 |
|  | **30-365 days ago** | 90 | 33.55 | 1.04 (0.64-1.70) | 0.87 | 0.83 (0.50-1.35) | 0.45 |
| **Addison’s disease** | **Unexposed** | 71 | 56.03 | Ref. |  | Ref. |  |
|  | **<30 days** | 6 | 6.05 | 0.53 (0.21-1.36) | 0.19 | 0.44 (0.17-1.15) | 0.09 |
|  | **30-365 days ago** | 53 | 33.57 | 1.33 (0.75-2.36) | 0.32 | 1.09 (0.58-2.03) | 0.79 |
| **Gastrointestinal outcomes** | | | | | | | |
| **Crohn’s disease** | **Unexposed** | 1258 | 55.80 | Ref. |  | Ref. |  |
|  | **<30 days** | 171 | 6.02 | 1.56 (1.27-1.92) | 0.00 | 1.57 (1.26-1.94) | 0.00 |
|  | **30-365 days ago** | 803 | 33.41 | 1.24 (1.06-1.45) | 0.01 | 1.24 (1.05-1.47) | 0.01 |
| **Ulcerative colitis** | **Unexposed** | 1995 | 55.67 | Ref. |  | Ref. |  |
|  | **<30 days** | 230 | 6.01 | 1.25 (1.04-1.49) | 0.01 | 1.29 (1.07-1.55) | 0.01 |
|  | **30-365 days ago** | 1331 | 33.32 | 1.38 (1.21-1.57) | 0.00 | 1.42 (1.24-1.63) | 0.00 |
| **Celiac Disease** | **Unexposed** | 764 | 55.88 | Ref. |  | Ref. |  |
|  | **<30 days** | 98 | 6.04 | 1.46 (1.12-1.91) | 0.00 | 1.42 (1.09-1.86) | 0.01 |
|  | **30-365 days ago** | 506 | 33.46 | 1.44 (1.19-1.74) | 0.00 | 1.37 (1.12-1.67) | 0.00 |
| **Autoimmune hepatitis** | **Unexposed** | 179 | 56.02 | Ref. |  | Ref. |  |
|  | **<30 days** | 13 | 6.05 | 1.04 (0.51-2.10) | 0.92 | 0.76 (0.37-1.55) | 0.44 |
|  | **30-365 days ago** | 132 | 33.56 | 2.63 (1.61-4.28) | 0.00 | 1.86 (1.11-3.11) | 0.02 |
| **Hematologic outcomes** | | | | | | | |
| **Purpura, thrombocytopenia including Henoch–Schönlein purpura (HSP)** | **Unexposed** | 824 | 55.92 | Ref. |  | Ref. |  |
|  | **<30 days** | 73 | 6.04 | 1.03 (0.75-1.41) | 0.86 | 0.92 (0.67-1.26) | 0.62 |
|  | **30-365 days ago** | 511 | 33.50 | 1.60 (1.27-2.02) | 0.00 | 1.41 (1.12-1.78) | 0.00 |
| **Idiopathic thrombocytopenic purpura (ITP)** | **Unexposed** | 181 | 56.03 | Ref. |  | Ref. |  |
|  | **<30 days** | 17 | 6.05 | 0.92 (0.48-1.75) | 0.79 | 0.88 (0.46-1.67) | 0.69 |
|  | **30-365 days ago** | 125 | 33.57 | 1.52 (0.96-2.40) | 0.07 | 1.41 (0.89-2.25) | 0.15 |
| **Agranulocytosis** | **Unexposed** | 683 | 55.94 | Ref. |  | Ref. |  |
|  | **<30 days** | 79 | 6.04 | 1.39 (1.01-1.93) | 0.04 | 1.24 (0.90-1.71) | 0.19 |
|  | **30-365 days ago** | 473 | 33.51 | 1.67 (1.31-2.14) | 0.00 | 1.43 (1.12-1.83) | 0.00 |
| **Haemolytic anaemia, including Hemolytic uremic syndrome (HUS)** | **Unexposed** | 8 | 56.05 | Ref. |  | Ref. |  |
|  | **<30 days** | 0 | 6.06 | Na | Na | Na | Na |
|  | **30-365 days ago** | 11 | 33.58 | 0.94 (0.19-4.71) | 0.94 | 0.83 (0.16-4.26) | 0.83 |
| **Neurologic outcomes** | | | | | | | |
| **Multiple sclerosis** | **Unexposed** | 1142 | 55.86 | Ref. |  | Ref. |  |
|  | **<30 days** | 123 | 6.03 | 1.14 (0.89-1.46) | 0.29 | 1.08 (0.84-1.40) | 0.53 |
|  | **30-365 days ago** | 676 | 33.44 | 1.16 (0.98-1.38) | 0.09 | 1.09 (0.91-1.31) | 0.35 |
| **Demyelinating disease of the central nervous system** | **Unexposed** | 216 | 56.01 | Ref. |  | Ref. |  |
|  | **<30 days** | 24 | 6.05 | 1.46 (0.82-2.58) |  | 1.34 (0.75-2.39) | 0.32 |
|  | **30-365 days ago** | 161 | 33.55 | 1.41 (0.94-2.11) |  | 1.29 (0.86-1.94) | 0.22 |
| **Acute disseminated encephalomyelitis (ADEM)** | **Unexposed** | Na | Na | Ref. |  | Ref. |  |
|  | **<30 days** | Na | Na | Na | Na | Na | Na |
|  | **30-365 days ago** | Na | Na | Na | Na | Na | Na |
| **Bell's palsy** | **Unexposed** | 442 | 55.95 | Ref. |  | Ref. |  |
|  | **<30 days** | 69 | 6.04 | 1.74 (1.21-2.51) | 0.00 | 1.59 (1.10-2.30) | 0.01 |
|  | **30-365 days ago** | 338 | 33.51 | 1.74 (1.32-2.29) | 0.00 | 1.56 (1.18-2.06) | 0.00 |
| **Narcolepsy** | **Unexposed** | 71 | 56.03 | Ref. |  | Ref. |  |
|  | **<30 days** | 4 | 6.05 | 0.31 (0.09-1.06) | 0.06 | 0.28 (0.08-1.07) | 0.06 |
|  | **30-365 days ago** | 27 | 33.57 | 0.58 (0.28-1.23) | 0.15 | 0.57 (0.26-1.28) | 0.18 |
| **Polyneuropathy** | **Unexposed** | 803 | 55.84 | Ref. |  | Ref. |  |
|  | **<30 days** | 101 | 6.03 | 2.06 (1.55-2.73) | 0.00 | 1.51 (1.13-2.00) | 0.00 |
|  | **30-365 days ago** | 502 | 33.44 | 2.31 (1.83-2.91) | 0.00 | 1.66 (1.31-2.11) | 0.00 |
| **Myasthenia gravis** | **Unexposed** | 69 | 56.04 | Ref. |  | Ref. |  |
|  | **<30 days** | 8 | 6.05 | 0.98 (0.39-2.46) | 0.96 | 0.86 (0.33-2.21) | 0.75 |
|  | **30-365 days ago** | 67 | 33.57 | 1.79 (0.87-3.66) | 0.11 | 1.58 (0.75-3.33) | 0.23 |
| **Rheumatologic outcomes** | | | | | | | |
| **Rheumatoid arthritis** | **Unexposed** | 1460 | 55.80 | Ref. |  | Ref. |  |
|  | **<30 days** | 172 | 6.02 | 1.63 (1.32-2.02) | 0.00 | 1.22 (0.99-1.52) | 0.07 |
|  | **30-365 days ago** | 963 | 33.42 | 1.75 (1.49-2.07) | 0.00 | 1.30 (1.10-1.54) | 0.00 |
| **Reactive/idiopathic inflammatory arthritis** | **Unexposed** | 194 | 56.01 | Ref. |  | Ref. |  |
|  | **<30 days** | 13 | 6.05 | 0.51 (0.26-1.01) | 0.05 | 0.50 (0.25-1.03) | 0.06 |
|  | **30-365 days ago** | 104 | 33.55 | 0.81 (0.55-1.21) | 0.30 | 0.81 (0.52-1.25) | 0.34 |
| **Arthralgia** | **Unexposed** | 7182 | 54.07 | Ref. |  | Ref. |  |
|  | **<30 days** | 827 | 5.82 | 1.24 (1.13-1.36) | 0.00 | 1.11 (1.01-1.21) | 0.03 |
|  | **30-365 days ago** | 5130 | 32.27 | 1.32 (1.24-1.41) | 0.00 | 1.18 (1.11-1.26) | 0.00 |
| **Connective tissue diseases** | **Unexposed** | 614 | 55.89 | Ref. |  | Ref. |  |
|  | **<30 days** | 74 | 6.03 | 1.50 (1.08-2.07) | 0.02 | 1.21 (0.87-1.67) | 0.25 |
|  | **30-365 days ago** | 364 | 33.47 | 1.29 (1.02-1.64) | 0.04 | 1.02 (0.80-1.30) | 0.87 |
| **Dermatopolymyositis** | **Unexposed** | 54 | 56.04 | Ref. |  | Ref. |  |
|  | **<30 days** | 7 | 6.05 | 1.22 (0.41-3.62) | 0.72 | 1.09 (0.35-3.34) | 0.88 |
|  | **30-365 days ago** | 47 | 33.57 | 1.59 (0.86-2.95) | 0.14 | 1.38 (0.74-2.58) | 0.31 |
| **Sjogren’s syndrome** | **Unexposed** | 313 | 55.95 | Ref. |  | Ref. |  |
|  | **<30 days** | 38 | 6.04 | 1.55 (0.98-2.43) | 0.06 | 1.22 (0.78-1.89) | 0.39 |
|  | **30-365 days ago** | 177 | 33.51 | 1.23 (0.87-1.73) | 0.23 | 0.95 (0.68-1.34) | 0.79 |
| **SLE (Lupus)** | **Unexposed** | 145 | 56.01 | Ref. |  | Ref. |  |
|  | **<30 days** | 16 | 6.05 | 1.38 (0.67-2.86) | 0.38 | 1.28 (0.62-2.67) | 0.50 |
|  | **30-365 days ago** | 80 | 33.55 | 1.25 (0.75-2.09) | 0.39 | 1.11 (0.65-1.91) | 0.69 |
| **Systemic sclerosis** | **Unexposed** | 120 | 56.03 | Ref. |  | Ref. |  |
|  | **<30 days** | 13 | 6.05 | 1.41 (0.66-3.03) | 0.38 | 0.99 (0.47-2.10) | 0.97 |
|  | **30-365 days ago** | 59 | 33.57 | 1.05 (0.58-1.90) | 0.87 | 0.67 (0.37-1.22) | 0.19 |

**Supplemental table 11. Overview of published literature supporting an association or no association between autoimmune mediated diseases and SARS-CoV-2 infection and COVID-19 mRNA vaccination. Last updated 20. Dec 2024.**

| Condition | Infection | | | Vaccination | | |
| --- | --- | --- | --- | --- | --- | --- |
|  | Reduced risk | No association | Increased risk | Reduced risk | No association | Increased risk |
| **Neurologic outcomes** |  |  |  |  |  |  |
| Multiple sclerosis |  | [1^*^, 2] | [1,^*^ 3, 4, 5] |  | [5] |  |
| Demyelinating disease of the central nervous system |  |  |  |  |  |  |
| Acute disseminated encephalomyelitis (ADEM) |  |  |  |  | [6, 7] | [8] |
| Bell’s palsy |  |  | [9-12] | [10] | [9, 13-16] | [8, 17-20] |
| Narcolepsy |  |  | [21] |  |  | [7] |
| Polyneuropathy |  |  |  |  |  |  |
| Myasthenia gravis |  | [3, 22] | [5, 9] |  | [9, 23] | [5] |
| **Rheumatologic outcomes** |  | [24] | [25] |  |  |  |
| Rheumatoid arthritis (RA) |  | [13, 22, 26, 27] | [3, 4, 14, 28-30] | [7, 31] | [32, 33] | [29]^#^ |
| Reactive/idiopathic inflammatory arthritis |  | [26] |  |  | [7] |  |
| Arthralgia |  |  |  |  |  | [34] ^†^ |
| Connective tissue diseases |  |  | [14] |  | [32] |  |
| Dermatopolymyositis |  | [4, 13, 26, 27] | [14] |  | [32] |  |
| Sjogren’s syndrome |  | [4, 13, 22, 26, 27] | [3, 14, 30] |  | [7, 31, 32] |  |
| Systemic lupus erythematosus (SLE) | [13, 26] | [3, 4, 22, 27] | [14, 30] | [7] | [32] | [31] |
| Scleroderma |  | [3, 4, 13, 26] | [14] |  | [32] |  |
| **Endocrinologic outcomes** |  |  |  |  |  |  |
| Hyperthyroidism |  | [35, 36] | [37] |  | [38] |  |
| Graves’ Disease | [26] |  | [3, 4] |  | [38] |  |
| Thyroiditis |  | [27, 36] |  |  | [38] |  |
| Hashimoto thyroiditis | [26] | [4, 22, 33] | [3] |  |  |  |
| Addison’s disease |  |  |  |  |  |  |
| **Gastrointestinal outcomes** |  |  |  |  |  |  |
| Crohn’s disease | [26] | [4^‡^, 33^‡^] | [3, 13, 14^‡^, 22^‡^, 30, 39^‡^] |  | [31, 32] | [33] ^§^ |
| Ulcerative colitis (UC) |  | [4^‡^, 13, 33^‡^] | [3, 14^‡^, 22^‡^, 26, 30, 39^‡^] |  | [31, 32] | [33] ^§^ |
| Celiac disease |  | [22, 26, 33, 40, 41] | [3] |  |  |  |
| Autoimmune hepatitis |  | [3, 26] |  |  | [42] |  |
| **Dermatologic outcomes** |  |  |  |  |  |  |
| Urticaria |  |  | [43] |  |  |  |
| Erythema multiforme including Stevens–Johnson syndrome |  |  | [44, 45] |  |  | [45] |
| Erythema nodosum |  |  |  |  |  |  |
| Alopecia |  | [46] | [3, 13, 30, 47] |  | [31, 32] |  |
| **Hematologic outcomes** |  |  |  |  |  |  |
| Purpura, thrombocytopenia including Henoch–Schönlein purpura (HSP) |  |  | [48, 49] |  | [48, 50-52] | [49] |
| Idiopathic thrombocytopenic purpura (ITP) |  |  | [3] |  | [7, 50-52] | [8]^ǁ^ |
| Agranulocytosis |  |  |  |  |  |  |
| Haemolytic anaemia, including Hemolytic uremic syndrome (HUS) |  |  |  |  |  |  |
| * Significant for COVID-19 hospitalized but not for all COVID-19 ^†^ Studied among RA patients  ^‡^ Reporting on Inflammatory bowel disease  ^§^ Reporting on Inflammatory bowel disease, only certain age groups are significant, and results did not hold up to multiple testing  ^ǁ^ Only after first dose of Comirnaty, but not after subsequent doses  ^#^ Relying on a self-reporting through a survey | | | | | | |

References

1. Montgomery, S., et al., *SARS-CoV-2 infection and risk of subsequent demyelinating diseases: national register-based cohort study.* Brain Commun, 2024. **6**(6): p. fcae406.

2. Zarifkar, P., et al., *Frequency of Neurological Diseases After COVID-19, Influenza A/B and Bacterial Pneumonia.* Front Neurol, 2022. **13**: p. 904796.

3. Tesch, F., et al., *Incident autoimmune diseases in association with SARS-CoV-2 infection: a matched cohort study.* Clinical Rheumatology, 2023. **42**(10): p. 2905-2914.

4. Peng, K., et al., *Risk of autoimmune diseases following COVID-19 and the potential protective effect from vaccination: a population-based cohort study.* eClinicalMedicine, 2023. **63**.

5. Salmaggi, A., et al., *Impact of COVID-19 disease and COVID-19 vaccinations on hospital admissions for neurological diseases in the Lombardia over-12 population. Data from a self-controlled case series analysis.* Neurol Sci, 2024.

6. Stowe, J., J. Lopez-Bernal, and N. Andrews, *The risk of acute disseminated encephalomyelitis (ADEM) following covid-19 vaccination in England: A self-controlled case-series analysis.* Hum Vaccin Immunother, 2024. **20**(1): p. 2311969.

7. Li, X., et al., *Autoimmune conditions following mRNA (BNT162b2) and inactivated (CoronaVac) COVID-19 vaccination: A descriptive cohort study among 1.1 million vaccinated people in Hong Kong.* Journal of Autoimmunity, 2022. **130**: p. 102830.

8. Faksova, K., et al., *COVID-19 vaccines and adverse events of special interest: A multinational Global Vaccine Data Network (GVDN) cohort study of 99 million vaccinated individuals.* Vaccine, 2024. **42**(9): p. 2200-2211.

9. Patone, M., et al., *Neurological complications after first dose of COVID-19 vaccines and SARS-CoV-2 infection.* Nat Med, 2021. **27**(12): p. 2144-2153.

10. Li, X., et al., *Association between covid-19 vaccination, SARS-CoV-2 infection, and risk of immune mediated neurological events: population based cohort and self-controlled case series analysis.* Bmj, 2022. **376**: p. e068373.

11. Xu, E., Y. Xie, and Z. Al-Aly, *Long-term neurologic outcomes of COVID-19.* Nat Med, 2022. **28**(11): p. 2406-2415.

12. Kim, H.J., et al., *Risk of Bell's palsy following SARS-CoV-2 infection: a nationwide cohort study.* Clin Microbiol Infect, 2023. **29**(12): p. 1581-1586.

13. Lim, S.H., et al., *Autoimmune and Autoinflammatory Connective Tissue Disorders Following COVID-19.* JAMA Network Open, 2023. **6**(10): p. e2336120-e2336120.

14. Chang, R., et al., *Risk of autoimmune diseases in patients with COVID-19: a retrospective cohort study.* eClinicalMedicine, 2023. **56**.

15. Takeuchi, Y., et al., *A post-marketing safety assessment of COVID-19 mRNA vaccination for serious adverse outcomes using administrative claims data linked with vaccination registry in a city of Japan.* Vaccine, 2022. **40**(52): p. 7622-7630.

16. Walker, J.L., et al., *Safety of COVID-19 vaccination and acute neurological events: A self-controlled case series in England using the OpenSAFELY platform.* Vaccine, 2022. **40**(32): p. 4479-4487.

17. Baden, L.R., et al., *Efficacy and Safety of the mRNA-1273 SARS-CoV-2 Vaccine.* New England Journal of Medicine, 2021. **384**(5): p. 403-416.

18. *Vaccines and Related Biological Products Advisory Committee meeting. December 10, 2020. FDA briefing document. Pfizer BioNTech COVID-19 vaccine. [cited 2024 Apr 25]*.

19. *Vaccines and Related Biological Products Advisory Committee meeting. December 17, 2020. FDA briefing document. Moderna COVID-19 vaccine. [cited 2024 Apr 25]*.

20. Shibli, R., et al., *Association between vaccination with the BNT162b2 mRNA COVID-19 vaccine and Bell's palsy: a population-based study.* The Lancet Regional Health – Europe, 2021. **11**.

21. Voss, E.A., et al., *Contextualising adverse events of special interest to characterise the baseline incidence rates in 24 million patients with COVID-19 across 26 databases: a multinational retrospective cohort study.* EClinicalMedicine, 2023. **58**: p. 101932.

22. Syed, U., et al., *Incidence of immune-mediated inflammatory diseases following COVID-19: a matched cohort study in UK primary care.* BMC Medicine, 2023. **21**(1): p. 363.

23. Arbel, A., et al., *Association between COVID-19 vaccination and myasthenia gravis: A population-based, nested case-control study.* Eur J Neurol, 2023. **30**(12): p. 3868-3876.

24. Lee, S.W., et al., *Incidence if autoimmune inflammatory rheumatic diseases after COVID-19 in South Korea: A nationwide cohort study based on health insurance data.* International Journal of Rheumatic Diseases, 2024. **27**(6): p. e15219.

25. Kim, M.S., et al., *Long-Term Autoimmune Inflammatory Rheumatic Outcomes of COVID-19 : A Binational Cohort Study.* Ann Intern Med, 2024. **177**(3): p. 291-302.

26. Hileman, C.O., et al., *New-onset autoimmune disease after COVID-19.* Frontiers in Immunology, 2024. **15**.

27. Wee, L.E., et al., *Autoimmune Sequelae After Delta or Omicron Variant SARS-CoV-2 Infection in a Highly Vaccinated Cohort.* JAMA Netw Open, 2024. **7**(8): p. e2430983.

28. Marín, J.S., et al., *Increased incidence of rheumatoid arthritis after COVID-19.* Autoimmunity Reviews, 2023. **22**(10): p. 103409.

29. Al-Hawamdeh, M.I., et al., *Association between COVID-19 vaccines and development of chronic morbidities: a cross-sectional study in the Jordanian population.* Current Medical Research and Opinion, 2024. **40**(3): p. 537-543.

30. Heo, Y.-W., et al., *Long-Term Risk of Autoimmune and Autoinflammatory Connective Tissue Disorders Following COVID-19.* JAMA Dermatology, 2024. **160**(12): p. 1278-1287.

31. Jung, S.-W., et al., *Long-term risk of autoimmune diseases after mRNA-based SARS-CoV2 vaccination in a Korean, nationwide, population-based cohort study.* Nature Communications, 2024. **15**(1): p. 6181.

32. Ju, H.J., et al., *Risk of autoimmune skin and connective tissue disorders after mRNA-based COVID-19 vaccination.* Journal of the American Academy of Dermatology, 2023. **89**(4): p. 685-693.

33. Shani, M., et al., *The association between BNT162b2 vaccinations and incidence of immune-mediated comorbidities.* Vaccine, 2024. **42**(18): p. 3830-3837.

34. Takatani, A., et al., *Impact of SARS-CoV-2 mRNA vaccine on arthritis condition in rheumatoid arthritis.* Front Immunol, 2023. **14**: p. 1256655.

35. Zhang, Q., et al., *Thyroid dysfunction in the wake of Omicron: understanding its role in COVID-19 severity and mortality.* Front Endocrinol (Lausanne), 2024. **15**: p. 1412320.

36. Lui, D.T.W., et al., *Risk of Incident Thyroid Dysfunction in the Post-Acute Phase of COVID-19: A Population-Based Cohort Study in Hong Kong.* Endocrine practice : official journal of the American College of Endocrinology and the American Association of Clinical Endocrinologists, 2024. **30**(6): p. 528-536.

37. Huang, L.A., et al., *Association of COVID-19 Infection with Subsequent Thyroid Dysfunction: An International Population-Based Propensity Score Matched Analysis.* Thyroid, 2024. **34**(4): p. 442-449.

38. Bea, S., et al., *The Impact of COVID-19 Vaccination on Thyroid Disease in 7 Million Adult and 0.2 Million Adolescent Vaccine Recipients.* J Clin Endocrinol Metab, 2024.

39. Hadi, Y., et al., *Incidence, outcomes, and impact of COVID-19 on inflammatory bowel disease: propensity matched research network analysis.* Aliment Pharmacol Ther, 2022. **55**(2): p. 191-200.

40. Corrado, M.M., et al., *Previous SARS-CoV-2 Infection Is Not Associated With Increased Celiac Disease Autoimmunity in Children and Adolescents.* Official journal of the American College of Gastroenterology | ACG, 2023. **118**(9): p. 1698-1700.

41. Lexner, J., Y. Lindroth, and K. Sjöberg, *The risk for celiac disease after Covid-19 infection.* BMC Gastroenterol, 2023. **23**(1): p. 174.

42. Ng, A.J.J., et al., *Acute autoimmune hepatitis following COVID-19 mRNA vaccination: A population-based study using electronic health records in Singapore.* Vaccine, 2024. **42**(26): p. 126462.

43. Lee, S., et al., *Risks of chronic urticaria after SARS-CoV-2 infection: Binational population-based cohort studies from South Korea and Japan.* The Journal of Allergy and Clinical Immunology: In Practice, 2024. **12**(9): p. 2540-2542.e3.

44. Curtis, K.K., et al., *COVID-19 infection increases the development of Stevens-Johnson syndrome and toxic epidermal necrolysis: A retrospective cohort analysis.* Journal of the American Academy of Dermatology, 2024. **90**(6): p. 1300-1302.

45. Saleh, W., H. Alharbi, and S. Cha, *Increased prevalence of erythema multiforme in patients with COVID-19 infection or vaccination.* Scientific Reports, 2024. **14**(1): p. 2801.

46. Kim, J., et al., *Lack of Evidence of COVID-19 Being a Risk Factor of Alopecia Areata: Results of a National Cohort Study in South Korea.* Front Med (Lausanne), 2021. **8**: p. 758069.

47. Kim, J.-S., et al., *Risk of Alopecia Areata After COVID-19.* JAMA Dermatology, 2024. **160**(2): p. 232-235.

48. Hippisley-Cox, J., et al., *Risk of thrombocytopenia and thromboembolism after covid-19 vaccination and SARS-CoV-2 positive testing: self-controlled case series study.* Bmj, 2021. **374**: p. n1931.

49. Burn, E., et al., *Thrombosis and thrombocytopenia after vaccination against and infection with SARS-CoV-2 in Catalonia, Spain.* Nature Communications, 2022. **13**(1): p. 7169.

50. Simpson, C.R., et al., *First-dose ChAdOx1 and BNT162b2 COVID-19 vaccines and thrombocytopenic, thromboembolic and hemorrhagic events in Scotland.* Nature Medicine, 2021. **27**(7): p. 1290-1297.

51. Simpson, C.R., et al., *Second-dose ChAdOx1 and BNT162b2 COVID-19 vaccines and thrombocytopenic, thromboembolic and hemorrhagic events in Scotland.* Nat Commun, 2022. **13**(1): p. 4800.

52. Joy, M., et al., *Thrombocytopenic, thromboembolic and haemorrhagic events following second dose with BNT162b2 and ChAdOx1: self-controlled case series analysis of the English national sentinel cohort.* The Lancet Regional Health - Europe, 2023. **32**: p. 100681.
